# The economics of vision impairment and its leading causes: A systematic review

**DOI:** 10.1016/j.eclinm.2022.101354

**Published:** 2022-03-22

**Authors:** Ana Patricia Marques, Jacqueline Ramke, John Cairns, Thomas Butt, Justine H. Zhang, Iain Jones, Marty Jovic, Allyala Nandakumar, Hannah Faal, Hugh Taylor, Andrew Bastawrous, Tasanee Braithwaite, Serge Resnikoff, Peng T. Khaw, Rupert Bourne, Iris Gordon, Kevin Frick, Matthew J. Burton

**Affiliations:** a International Centre for Eye Health, London School of Hygiene and Tropical Medicine, London WC1E 7HT, United Kingdom; b School of Optometry and Vision Science, University of Auckland, Auckland, New Zealand; c University College London, London, United Kingdom; d Royal Free Hospital, London, United Kingdom; e Sightsavers, Haywards Heath, United Kingdom; f PricewaterhouseCoopers, Sydney, Australia; g Heller School for Social Policy and Management, Brandeis University, Waltham, MA, United States; h Department of Ophthalmology, University of Calabar, Calabar, Nigeria; i Africa Vision Research Institute, Durban, Kwa-Zulu Natal, South Africa; j Melbourne School of Population and Global Health, The University of Melbourne, Melbourne, Australia; k The Medical Eye Unit, Guy's and St Thomas' Hospital, London, United Kingdom; l School of Immunology and Microbiology and School of Life Course Sciences, Kings College, London, United Kingdom; m Brien Holden Vision Institute and SOVS, University of New South Wales, Sydney, NSW, Australia; n National Institute for Health Research Biomedical Research Centre, Moorfields Eye Hospital NHS Foundation Trust and UCL Institute of Ophthalmology, London, United Kingdom; o Vision and Eye Research Institute, School of Medicine, Anglia Ruskin University, Cambridge, United Kingdom; p Johns Hopkins Carey Business School, Baltimore, MD, United States

**Keywords:** Ophthalmology, Public health, Health economics, Systematic review, VI, Vision Impairment, AMD, Age- related macular degeneration, LMICs, Low Middle Income Countries, QALYs, Quality Adjusted Life Years, DALYs, Disability Adjusted Life Years, GBD, Global Burden of Disease, USD, United States Dollars ($), NR, Not reported, RE, Refractive Error, DR, Diabetic Retinopathy, EU, European, PPP, Purchasing power parity, MSVI, Moderate and Severe Vision Impairment, WHO, World Health Organization, ICD 11, International Statistical Classification of Diseases, Injuries and Causes of Death 11th revision, anti-VEGF, antivascular endothelial growth factor

## Abstract

Vision impairment (VI) can have wide ranging economic impact on individuals, households, and health systems. The aim of this systematic review was to describe and summarise the costs associated with VI and its major causes. We searched MEDLINE (16 November 2019), National Health Service Economic Evaluation Database, the Database of Abstracts of Reviews of Effects and the Health Technology Assessment database (12 December 2019) for partial or full economic evaluation studies, published between 1 January 2000 and the search dates, reporting cost data for participants with VI due to an unspecified cause or one of the seven leading causes globally: cataract, uncorrected refractive error, diabetic retinopathy, glaucoma, age-related macular degeneration, corneal opacity, trachoma. The search was repeated on 20 January 2022 to identify studies published since our initial search. Included studies were quality appraised using the British Medical Journal Checklist for economic submissions adapted for cost of illness studies. Results were synthesized in a structured narrative. Of the 138 included studies, 38 reported cost estimates for VI due to an unspecified cause and 100 reported costs for one of the leading causes. These 138 studies provided 155 regional cost estimates. Fourteen studies reported global data; 103/155 (66%) regional estimates were from high-income countries. Costs were most commonly reported using a societal (*n* = 48) or healthcare system perspective (*n* = 25). Most studies included only a limited number of cost components. Large variations in methodology and reporting across studies meant cost estimates varied considerably. The average quality assessment score was 78% (range 35–100%); the most common weaknesses were the lack of sensitivity analysis and insufficient disaggregation of costs. There was substantial variation across studies in average treatment costs per patient for most conditions, including refractive error correction (range $12–$201 ppp), cataract surgery (range $54–$3654 ppp), glaucoma (range $351–$1354 ppp) and AMD (range $2209–$7524 ppp). Future cost estimates of the economic burden of VI and its major causes will be improved by the development and adoption of a reference case for eye health. This could then be used in regular studies, particularly in countries with data gaps, including low- and middle-income countries in Asia, Eastern Europe, Oceania, Latin America and sub-Saharan Africa.

## Introduction

Vision impairment (VI) is a problem for a large and growing number of people globally. In 2020 an estimated 1.1 billion people were living with VI, and this is projected to increase to 1.8 billion people in 2050.^1^ About 90% of those affected live in low- and middle-income countries (LMICs).^2^ VI and other eye health problems have a profound impact on individuals, households, health systems, social development and the economy.^2^, ^3^, ^4^, ^5^

VI is associated with considerable economic costs. We recently reported a new estimate for global annual economic productivity loss associated with VI of US$ 411 billion for 2020.^6^ In addition to this loss of economic productivity, there are costs to the health system to provide and individuals to access eye care, and other costs related to complications of vision loss and its effects on comorbid conditions such as depression, cardiovascular diseases, diabetes and hypertension.^7^^,^^8^ Access to eye care services and the associated costs should be a topic of concern for governments due to population aging and the expansion of expensive medical technologies placing significant pressure on healthcare delivery systems.^9^^,^^10^

A systematic review published in 2013 identified 22 studies that reported costs associated with VI from the main causes of VI in high-income countries.^11^ Here we report a global systematic review in which we describe and summarise the costs associated with VI and its major causes. We have undertaken this review for three main reasons. First, we expanded the search to include low- and middle-income countries to provide a more global picture. Second, we expanded the search to include the seven major causes of VI identified in the 2015 global prevalence estimates—cataract, uncorrected refractive error, diabetic retinopathy, glaucoma, age-related macular degeneration (AMD), corneal opacity and trachoma.^12^ Finally, new treatments (e.g. anti-VEGF treatment) have commenced or expanded, which may result in substantial costs or savings, and are thus likely to affect the societal cost of VI.

## Methods

### Protocol and registration

The protocol for this systematic review was registered on Open Science Framework (https://osf.io/9au3w - doi10.17605/OSF.IO/6F8VM) and published.^13^

### Search strategy and selection criteria

A literature search was performed in MEDLINE (Ovid) on 16 November 2019 and the Centre for Reviews and Dissemination (CRD) database (which includes the National Health Service Economic Evaluation Database (NHS EED), the Database of Abstracts of Reviews of Effects (DARE) and the Health Technology Assessment (HTA) database) on 12 December 2019. On 20 January 2022 we repeated the search to identify studies published since our initial search. The search strategy was constructed by an information specialist (IG) (supplementary data Appendix 1 p 1) and was provided as an Annex to our protocol.^13^ No language or geographical restriction was applied. To ensure contemporary estimates were identified, the search was restricted to papers published between 1 January 2000 and the search dates. The references of all included studies were reviewed for additional potentially relevant studies. We also provided the list of included studies to field experts, these being health economists and eye care researchers who have conducted economic evaluation in eye care, to identify further potentially relevant studies and reports in the grey literature. The inclusion criteria are summarized in Table 1.

**Table 1:** Summary of the PICOS elements for the systematic review of studies reporting costs associated with VI and its major causes.

Participants	*Included:* Participants with VI from an unspecified cause or due to one of the leading causes of VI globally (i.e. cataract, uncorrected refractive error, diabetic retinopathy, glaucoma, AMD, corneal opacity or trachoma).^12^
*Interventions*	*Included:* Studies reporting services for cataract were included regardless of VI as this tends to be a one-off intervention and the cost of treatment does not vary with the severity of vision loss. Studies reporting services for refractive error were included regardless of VI as these tend to be a series of irregular one-off efficacious interventions. *Excluded:* Studies that reported costs of screening or treatment services for the remaining causes of VI that did not report costs for people with VI.
*Comparators*	Not relevant
*Outcomes*	*Included:* Studies reporting any of the following outcomes among people with VI: Direct costs, indirect costs, productivity losses (e.g. absenteeism costs, lost work days, employment opportunities), informal care (e.g. caregivers costs, number of caregivers hours), intangible costs (e.g. QALYs, DALYs), transfer payments or deadweight losses. *Excluded:* Studies that only reported incremental costs, net costs, incremental benefits or net benefits, incremental cost effectiveness ratio, incremental cost benefit ratios without also reporting actual costs.
*Study Design*	*Included:* Partial economic evaluation studies such as cost of illness studies, burden of illness/diseases and full economic evaluation studies such as cost-effectiveness and cost-benefit studies. *Excluded:* Model-based economic evaluation studies not reporting any costs, primary data or based on reviews of existing studies.

VI: Vision impairment; AMD: Age-related macular degeneration; QALYs: Quality adjusted life years; DALYs: Disability adjusted life years.

Vision impairment is categorised based on visual acuity and visual field and varies by jurisdictions and countries. The 11th revision of the World Health Organization (WHO) International Statistical Classification of Diseases, Injuries and Causes of Death (ICD11) defines vision impairment based on presenting visual acuity in the better seeing eye: mild vision impairment is visual acuity worse than 6/12 to 6/18 inclusive, moderate to severe vision impairment (MSVI) is visual acuity worse than 6/18 to 3/60 inclusive and blindness is visual acuity worse than 3/60.^14^ Where these categories have not been used, we used (and reported) the categories defined in the primary studies. We use the term vision impairment inclusive of blindness and mild, moderate and severe vision impairment except for studies that only reported costs for persons with blindness where we used the word blindness in our results.

### Study selection

**Figure 1:**
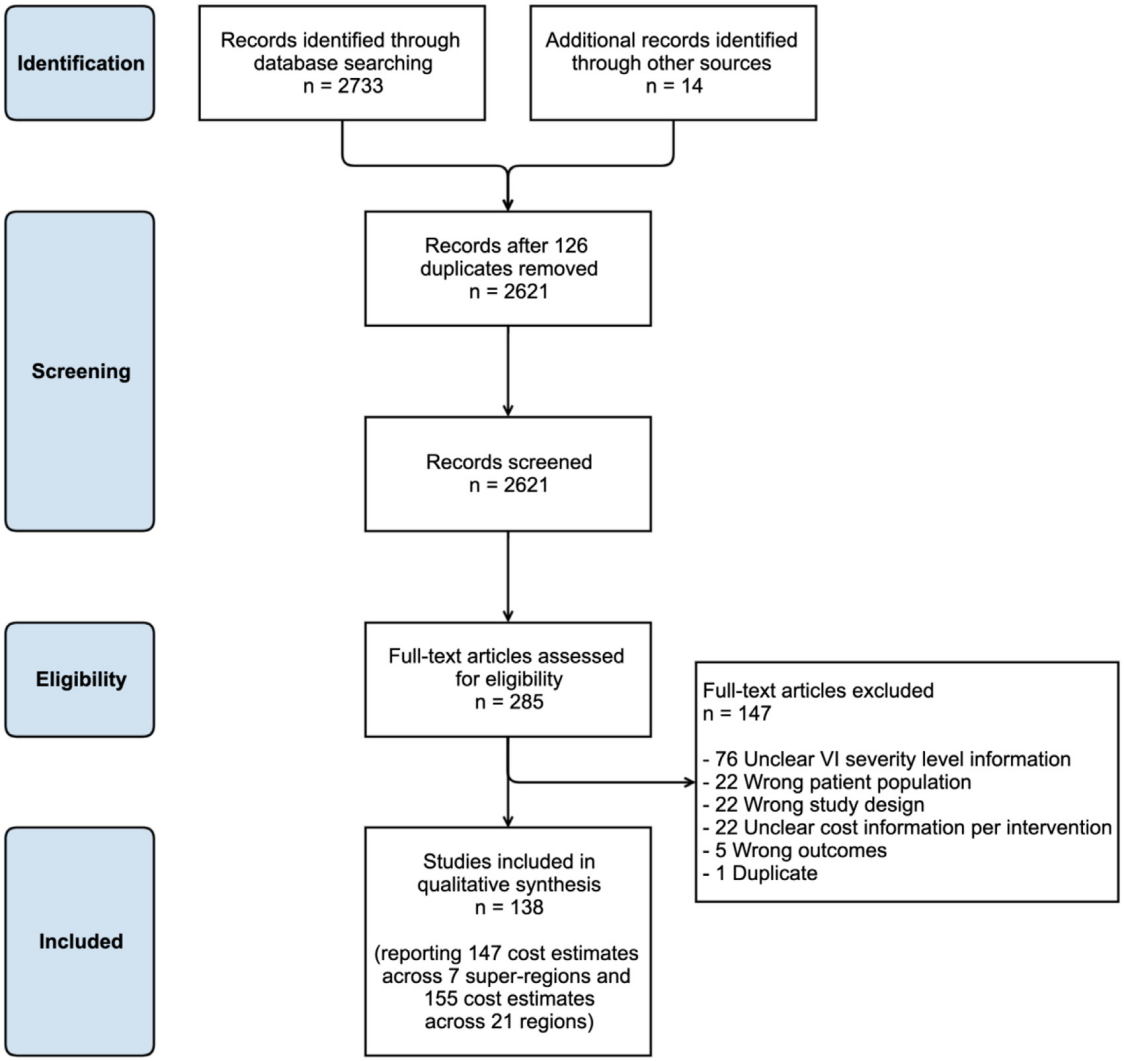
PRISMA flow diagram.

All titles and abstracts were screened by two investigators independently (APM and one of JR, JZ or ThB) in Covidence systematic review software (Veritas Health Innovation, Melbourne, Australia; available at www.covidence.org). After completing the screening process, full texts were assessed by two investigators (APM and one of JR, JZ or ThB) independently to establish eligibility for inclusion into the study. Any conflict in relation to screening was discussed between the two investigators and resolved with a third investigator when necessary. The PRISMA flow diagram (Figure 1) outlines the search process and the reasons for study exclusion.

### Data extraction

APM extracted all relevant data which was then verified by one of ThB, JZ or JR. The items extracted included:

• *Study details:* study period, country/countries of study, age range of participants, study design (e.g. cost of illness, burden of illness/diseases, cost effectiveness or cost benefit studies);
• *Methodological details:* epidemiological approach (i.e. incidence or prevalence based), perspective of analysis (e.g. societal/government/ healthcare system/payer/healthcare provider or patient), method of resource quantification (e.g. top-down, bottom-up, combination), discounting methods (i.e. discount rate applied and justification);
• *Data and definitions:* main data sources (e.g. published expenditure reports, administrative database, population survey, patient clinical records, patient diaries, specially designed questionnaires, published literature), VI definition & severity (i.e. blind/ moderate or severe VI), cause of VI (and definition) if specified, disease stage if specified, currency in which costs were reported, year of cost data, cost estimates including direct costs, productivity costs, informal care costs, loss of well-being measures (e.g. intangible costs measured with quality-adjusted life years (QALYs), disability adjusted life years (DALYs), years of sight loss);
• *Analysis of uncertainty:* type of uncertainty analysed (parameter uncertainty, methodological uncertainty or modelling uncertainty), choice of parameters included in sensitivity analysis, method to analyse uncertainty (e.g. univariate sensitivity analysis, probabilistic sensitivity analysis).

### Quality assessment of studies

Formal international guidelines for quality assessment of economic studies are lacking, so to assess quality of included studies we used the British Medical Journal Checklist for economic submissions,^15^ adapted for cost of illness studies.^16^ All included studies were appraised by two investigators independently (APM and one of ThB, IJ, AN and MJ). The items assessed included whether the study: defined the disease; described the epidemiological approach; disaggregated the costs; described and assessed the data sources; adequately explained the methods; indicated the study perspective; described the resource utilization; explained the valuation of unit cost; presented and discussed the results; and performed sensitivity analysis to assess the robustness of their results.^15^^,^^16^ Each quality criterion was rated as one of: yes (1 point), partial (0.5 points), no (zero points), or not applicable (zero points, plus the item was removed from the denominator). A global score was calculated for each study, being the total number of points allocated as a proportion of the total points applicable for each study. Equal weight was assigned to each item of the checklist. We did not exclude any study based on its quality score. Studies with a higher score indicates higher quality.

### Synthesis of results

We divided the included studies into two groups: (1) ‘general VI studies’ that reported costs for people with VI (without specifying the cause), and (2) studies that reported costs for people with one of the seven specified causes of vision loss (Table 1).

Studies were then characterized in terms of country/countries of study (grouped by Global Burden of Disease (GBD) super-region [*n* = 7] and region [*n* = 21]), study design, perspective of analysis, epidemiological approach, type of reported costs, level of reporting, methods of resource quantification and methods to deal with uncertainty. If the epidemiological approach, perspective of analysis or study design was not stated, it was assigned by two investigators (APM and ThB) independently and finalised by consensus (more details in supplementary data Appendix 2 p 2-3).

To enhance the comparability of the data, costs reported for any year prior to 2018 were inflated to 2018 values using a country-specific gross domestic product deflator,^17^ and then converted to USD ($) purchasing power parities (ppp)^18^ to equalise the purchasing power of different currencies. Whenever the year of cost data was not reported, the year of publication was used as a proxy (more details in supplementary Appendix 2 p 2-3).

Cost components reported in each study are summarised in supplementary Table 1 (supplementary Appendix p 4–6). We compared all cost categories against a standard framework (supplementary data Appendix 2 p 2-3) and recategorized components where indicated to increase comparability between studies.

The process outlined by Mandrik et al. was followed to decide whether to combine studies.^19^ Costs were synthesized in a structured narrative way using seven summary tables: two for studies reporting global and national estimates and five tables reporting average costs. Costs were reported in four categories: direct costs, productivity costs, informal care costs and intangible costs. The unit of observation was reported for average costs and included costs per episode of care (for all services provided within a specified period of time such as medical appointments, tests and treatments), costs per patient (for all episodes of care provided in a specific period of time), costs per surgery and costs per spectacles. Whenever information was available, we classified direct costs as direct medical (e.g. inpatient care, outpatient care, medical prescriptions and medical examinations) or direct non-medical costs (e.g. home care, transport), and costs of productivity losses as morbidity-related (e.g. absenteeism, presenteeism, reduced workforce participation) or mortality-related productivity losses (i.e. productivity losses due to premature mortality). Intangible costs were reported in non-monetary measures, such as QALYs foregone and DALYs gained, because objective monetary valuation of intangible costs is controversial and there is no common acceptable value across countries^20^^,^^21^ (more details provided in supplementary data Appendix 2 p 2-3).

### Role of the funding source

The funders had no role in the study design, data collection, data analysis, data interpretation, writing of the manuscript, or in the decision to submit the manuscript for publication. APM and ThB had access to and verified the data reported in the manuscript. The corresponding author had full access to all of the data and the final responsibility to submit for publication following approval from all co-authors.

## Results

### Search results and study characteristics

A total of 2733 records were identified from the literature search and 14 unpublished reports were provided by field experts. After screening titles and abstracts, 285 articles underwent full text screening, and 138 studies met our criteria and were included in this analysis (Figure 1).

### Geographic distribution

Some of the included studies reported cost estimates in more than one super-region, region, country or eye condition. Five studies reported estimates for more than one super-region and seven studies reported estimates for more than one region, so the 138 studies provided 147 estimates across the seven GBD super-regions and 155 estimates across the 21 GBD regions (Figure 1; supplementary Table 2 p 7-8). Almost 1 in 10 estimates presented global data (14/138; 10%) and a further two-thirds reported results from the high-income super-region (96/147; 65%) (Table 2). Of the regional estimates, Western Europe (43/155; 28%) and North America (35/155; 23%) were most common. Eight of the 21 regions had no estimates (Central Asia, Eastern Europe, Southern Latin America, Caribbean, Andean Latin America, Oceania, Central sub-Saharan Africa and Southern sub-Saharan Africa) (supplementary Table 2 p 7-8).

**Table 2:** Distribution of 138 included studies reporting costs associated with VI and its major causes by super-region, study participants age-range, study design, perspective of analysis and epidemiological approach.

Studies characteristics	General VI studies		Condition-specific studies		Total	
	*n*	%	*n*	%	*n*	%
**Number of super-regions estimates (*n*** **=** **147)**^a^						
High Income	30	70%	66	63%	96	65%
South Asia	2	5%	8	8%	10	7%
Southeast Asia, East Asia, and Oceania	3	7%	5	5%	8	5%
Latin America and Caribbean	1	2%	6	6%	7	5%
Sub-Saharan Africa	1	2%	6	6%	7	5%
Central Europe, Eastern Europe, and Central Asia	1	2%	2	2%	3	2%
North Africa and Middle East	0	0%	2	2%	2	1%
Global	5	12%	9	9%	14	10%
**Study participants age-range (*n*** **=** **138)**						
All ages	21	55%	16	16%	37	27%
Youth, Adults and Seniors (all > 15 years)	16	42%	64	64%	80	58%
Children and Youth only (all < 20 years)	1	3%	3	3%	4	3%
Age range not stated	0	0%	17	17%	17	12%
**Study design (*n*** **=** **138)**						
Cost of illness study	28	74%	56	56%	84	61%
Cost analysis	4	11%	24	24%	28	20%
Cost effectiveness study	0	0%	17	17%	17	12%
Other b	6	16%	3	3%	9	7%
**Study perspective (*n*** **=** **138)**^c^						
Societal	21	55%	27	27%	48	35%
Healthcare system	4	11%	21	21%	25	18%
Third party payer	1	3%	22	22%	23	17%
Patient	7	18%	7	7%	14	10%
Hospital	0	0%	7	7%	7	5%
Other d	4	11%	2	2%	6	5%
Multiple e	0	0%	11	11%	11	8%
Not applicable f	1	3%	3	3%	4	3%
**Study epidemiological approach (*n*** **=** **138)**						
Prevalence-based	34	89%	90	90%	124	90%
Incidence-based	3	8%	5	5%	8	6%
Incidence and prevalence-based	0	0%	3	3%	3	2%
Not applicable f	1	3%	2	2%	3	2%

a Studies reported costs estimates in more than one super-region therefore the sum of studies distributed by super-region (*n* = 147) is greater than the number of studies (*n* = 138);

b Includes 4 case control studies, 2 case reports, 1 study reporting each of a method to collect personal costs, employment data and data on informal care;

c We assigned a study perspective in 52 studies when authors had not;

d Includes studies adopting a governmental (*n* = 4), caregiver (*n* = 1) and employer (*n* = 1) perspective;

e Includes economic evaluation results from 2 perspectives, most often (societal or healthcare system perspective together (*n* = 3) or combined with other perspectives (*n* = 6). Other combinations included patient perspective reported with other perspectives (*n* = 2);

f These studies reported an estimate of the impact of vision impairment on the labour market in terms of well-being and thus did not require a study perspective or an epidemiological approach.

### Conditions

One hundred studies provided condition specific costs, three-quarters of which reported cost estimates for one of AMD (33/100), cataract (28/100), or glaucoma (16/100) (supplementary Table 2 p 7-8).

### Study design

Included studies were primarily cost of illness (84/138; 61%) or cost analysis (28/138; 20%) studies; there were 17 cost-effectiveness studies (17/138; 12%), Table 2. The age range of included participants varied widely, although studies largely focused on adults (all participants were >15 years).

Most studies (124/138; 90%) took a prevalence-based rather than an incidence-based approach to estimating costs (Table 2). The perspective used to estimate costs was not stated in 52 studies (38%) and for analysis purposes the reviewers had to assign a study perspective. Approximately one-third (48/138; 35%) of studies reported costs using a societal perspective; and roughly equal proportions used a healthcare system (25/138; 18%) or a payer (23/138; 17%) perspective (Table 2).

### Quality assessment of studies

The average quality assessment score across studies was 78% (median 80%, range 35–100%), (supplementary Tables 3 p 9 and 4 p 10–12). The quality items most often met by studies were providing a full or partly adequate description of the methods (135/138 [fully 108, partially 27]) and presenting and discussing the results suitably (136/138 [fully 106, partially 30]). The two items on which studies were weakest were reporting of sensitivity analysis (47/137 [fully 46, partially 1]) and disaggregation of costs (98/132 [fully 78, partially 20]).

### Review results

#### Cost report characteristics

The 138 studies provided 202 cost estimates distributed across four cost components: direct costs, productivity loss costs, informal care costs and intangible costs (Table 3). The cost components most frequently reported were direct costs (115/202; 57%), followed by productivity losses (37/202; 18%). A minority of studies included combinations of costs (41/138; 30%), e.g. direct costs and informal care costs (8/138; 6%) or direct costs, productivity losses and informal care costs (7/138; 5%) (data not shown). A summary of the cost components included in each study is presented in supplementary Table 1 (supplementary Appendix p 4–6).

**Table 3:** Characteristics of costs reported by 138 included studies reporting costs associated with VI and its major causes.

	General VI studies		Condition-specific studies		Total	
	*n*	%	*n*	%	*n*	**%**
**Number of reported costs components (*n*** **=** **202)**^a^						
Direct costs	28	39%	87	66%	115	57%
Productivity loss costs	19	27%	18	14%	37	18%
Informal care costs	14	20%	16	12%	30	15%
Intangible costs	10	14%	10	8%	20	10%
**Method of resource quantification (*n*** **=** **138)**^b^						
Top down (population-level)	14	37%	10	10%	24	17%
Bottom up (person-based)	20	53%	83	83%	103	75%
Top down and bottom up	4	10%	4	4%	8	6%
Not applicable c	0	0%	3	3%	3	2%
**Level of reporting estimates (*n*** **=** **138)**^d^						
Projected to a population (e.g. region, country)	20	53%	19	19%	39	28%
Recruited sample (e.g. average cost per patient or per treatment, excess cost)	16	42%	80	80%	96	70%
Both	2	5%	1	1%	3	2%
**Use of discounting (*n*** **=** **138)**						
Yes	6	16%	21	21%	27	20%
No	0	0%	0	0	0	0%
Not applicable e	32	84%	79	79%	111	80%
**Method use to deal with uncertainty (*n*** **=** **138)**						
Sensitivity analysis	13	34%	28	28%	41	30%
None	25	66%	72	72%	97	70%

a Studies reported more than one cost component therefore the sum of studies distributed by type of cost reported (*n* = 202) is greater than the number of studies (*n* = 138);

b Top-down method uses aggregate expenditures by cost component while bottom-up method assigns costs to individuals with a specific disease or condition;

c Includes 1 study that examined the relationship between vision impairment from cataract with time use (including paid work), 1 study that described the burden (measured with EQ5D Health States) of bilateral age-related macular degeneration and 1 study that reported impact on caregivers measured in number of work days lost;

d Population estimates provide information about the costs incurred in a defined population (district country, subregion, global) during a specific period of time. Average cost estimates provide information about the cost per patient or per treatment incurred in a specific population during a specific period of time;

e Discounting is only applicable in studies that report costs and consequences for multiple years.

Costs were most commonly derived using bottom-up methods either alone (103/138; 75%) or in combination with top-down methods (8/138; 6%). Condition-specific studies used the bottom-up method (83/100; 83%) more frequently than general VI studies (20/38; 53%). Less than one-third of studies (41/138; 30%) used sensitivity analysis to explore parameter or methodological uncertainty. Discounting methods to account for cost or benefits not incurred in the same year were used in all studies requiring it (*n* = 27). Due to heterogeneity, we summarise results narratively.^19^

#### Global and national population eye health cost estimates

Of the 39 studies reporting costs projected to a global or national population (Tables 4 and 5), seven were from similar settings or by the same group of authors that used similar methodology and reported similar cost components (Table 4). For example, four Australian studies were similar, and resembled reports from Japan, Canada and the United Kingdom by the same authors.^22^, ^23^, ^24^, ^25^, ^26^, ^27^, ^28^, ^29^ When reporting the cost of VI and blindness these studies showed that most of the direct costs were direct medical costs (>50%) and that productivity losses were mainly morbidity-related (>97%) rather than mortality-related. Two other global cost-effectiveness studies reported direct costs for cataract surgery and trachoma surgery using similar methodology (Table 5). Providing the two most cost-effective interventions in both diseases would avert 14.5 million DALYs per year globally (trachoma 11 million; cataract 3.5 million) at a cost of $5.87 billion ppp.^30^^,^^31^ Findings from most other studies were less comparable.

**Table 4:** National population cost estimates for vision impairment and its major causes. Costs are in billion 2018 USD purchasing power parity.

Country	Ref.	Cause	Year of cost data	Population for which costs were projected (million)	Perspective of analysis	in billion 2018 USD ppp							Intangible Costs	Quality appraisal score
						Direct Costs			Productivity losses			Informal Care costs		
						Medical (1)	Non- medical (2)	Total (1) + (2)	Morbidity (3)	Mortality (4)	Total (3)+(4)			
**National estimates**														
**High-income Asia Pacific**														
Japan	^26^	All causesl a	2007	1.64	Societal	11.03	9.52	20.55	5.36	0.06	5.42	–	229,085 DALYS	9.5
Singapore	^92^	All causes	NR	NR	Patient	–	–	–	–	–	–	–	1828 DALYS b	7
Singapore	^93^	RE – Myopia	2011	2.08	Patient	–	–	0.849	–	–	–	–	–	8
Singapore	^94^	AMD	2015	0.12	Health System	0.18	–	–	–	–	–	–	–	8.5
**Australasia**														
Australia	^22^	All causes	2004	0.48	Societal	1.4	0.8	2.20	1.81	0.01	1.82	0.86	41,187 DALYS c	7.5
Australia	^23^	All causes	2009	0.58	Societal	1.7	1.0	2.69	1.84	0.05	1.89	0.2	58,157 DALYS	9
Australia	^24^	Glaucoma	2005	0.21	Societal	–	–	0.34	–	–	0.05	0.10	6972 DALYS	9
Australia	^25^	DR	2005	0.28	Societal	–	–	0.04	–	–	0.09	0.05	9629 DALYS	8
**Western Europe**														
Germany	^95^	All causes	2016	3.27	Societal	25.51	–	–	15.93	–	25.51	–	–	10
Germany	^96^	All causes	2004	0.73	Societal	–	14.48	–	–	–	–	–	–	7.5
United Kingdom	^29^	All causes	2013	1.93	Societal	2.96	2.28	5.24	3.84	0.003	3.85	3.57	219,106 DALYS	9
United Kingdom	^96^	All causes	2004	1.1	Societal	–	25.55	–	–	–	–	–	–	7.5
France	^96^	All causes	2004	1.27	Societal	–	16.35	–	–	–	–	–	–	7.5
Italy	^96^	All causes	2004	1.03	Societal	–	21.07	–	–	–	–	–	–	7.5
**High-income North America**														
Canada	^27^^,^^28^	All causes	2007	0.82	Societal	4.86	0.29	5.15	4.18	–	–	0.67	77,306 DALYS	8
United States	^97^	All causes	2004	NR	Societal	21.15	14.48	35.63	10.46	–	–	–	–	9.5
United States	^98^	All causes	2004	3.7	Societal	6.64	–	–	–	–	–	0.52	209,202 QALYS lost	8.5
United States	^99^	All causes	2010	2.15	Societal	16.63	2.04	18.67	13.94	–	–	0.68	215,000 QALYS lost	8.5
United States	^100^	Refractive Error	2000	11.26	Payer	–	5.53	–	–	–	–	–	–	7
**North Africa, Middle East**														
Iran	^101^	Refractive Error	2013	75.15	Societal	17.0	–	–	–	–	–	–	–	5
**South Asia**														
Pakistan	^102^	Unspecified	2003–04	0.62	Societal	–	–	–	0.57	–	–	–	–	5
**Multi-country estimates**														
28 EU countries	^40^	All causes	2014	11.27	Societal	–	–	–	39.3; 49.3;85.4 d	–	–	–	–	8.5
9 countries	^38^	All causes	2011	25.41	Societal	–	–	–	28.8; 75.57^e^	–	–	–	–	8.5

USD – United States Dollars ($); NR – Not reported; RE – Refractive Error; AMD - age-related macular degeneration; DR – Diabetic Retinopathy; DALYs – Disability Adjusted Life Years; QALYS – Quality Adjusted Life Years; EU – European; ppp- purchasing power parity;

a General VI study – all causes of VI;

b Annual QALYs loss per 100,000 persons due to VI;

c This DALYS estimate includes only years of life lived with disability leaving out of this estimate years of life lost due to premature mortality. DALYs estimates usually combine years of life lived with disability and life lost due to premature mortality;

d This study estimated productivity losses costs using three models, the minimum wage model ($39.3 billion ppp), the Gross Domestic Product Adjusted model ($49.3 billion ppp), and the Gross National Income model ($85.4 billion ppp);

e This study valued productivity losses costs using two models the minimum wage model ($28.8 billion ppp) and the Gross National Income model ($75.57 billion ppp).

**Table 5:** Global population cost estimates for vision impairment and its major causes. Costs are in billion 2018 USD purchasing power parity.

Country	Ref.	Cause	Year of cost data	Population for which costs were projected (million)	Perspective of analysis	In billion 2018 USD ppp							Intangible Costs	Quality appraisal score
						Direct Costs			Productivity losses			Informal Care costs		
						Medical (1)	Non- medical (2)	Total (1) + (2)	Morbidity (3)	Mortality (4)	Total (3)+(4)			
**Global estimates**														
World	^32^	All causes a	2010	733.0	Societal	2645.06	**–**	**–**	**–**	**–**	193.36	282.98	117.7 million DALYS	10
World	^33^	All causes	2000	25.0	Societal	**–**	**–**	**–**	26.87	**–**	**–**	**–**	–	8
World	^39^	All causes	2017	1077.1	Societal	**–**	**–**	**–**	381.06	**–**	**–**	**–**	**–**	7.5
World	^103^	Refractive Error	2007	158.5	Societal	**–**	**–**	**–**	321.38; 511.37 b	**–**	**–**	**–**	**–**	6.5
World	^34^	Refractive Error	2015	537.6	Societal	**–**	**–**	**–**	239.75;5.54 c	**–**	**–**	17.57;0.39 c	**–**	9
World	^37^	Refractive Error	2011	244	Societal	**–**	**–**	**–**	28.59	**–**	**–**	**–**	**–**	9
World	^30^	Cataract	2000	N.R	Societal	5.54^d^	**–**	**–**	**–**	**–**	**–**	**–**	3.5 million DALYS e	8
World	^31^	Trachoma	2000	N.R	Societal	0.33 f	**–**	**–**	**–**	**–**	**–**	**–**	11 million DALYS g	7.5
World	^36^	Trachoma	2003	146	Societal	**–**	**–**	**–**	7.10	**–**	**–**	0.54	**–**	7
World	^35^	Trachoma	1995	9.1	Societal	**–**	**–**	**–**	4.46	**–**	**–**	**–**	**–**	6
World	^41^	Trachoma	2005	N.R	Patient	**–**	**–**	**–**	0.78	**–**	**–**	**–**	**–**	9

USD – United States Dollars ($); NR – Not reported; DALYs – Disability Adjusted Life Years; ppp- purchasing power parity;.

a General VI study – all causes of VI;

b This study estimated productivity losses costs using two models, the Gross domestic Product Adjusted model ($321.38 billion ppp) and the Gross domestic Product Unadjusted model ($511.37 billion ppp);

c Productivity losses and informal care costs resulting from VI caused by uncorrected myopia (highest value) and myopic macular degeneration (lowest value);

d Cost of providing extra capsular cataract surgery at 80% coverage level;

e DALYS per year averted with extra capsular cataract surgery provided at 95% coverage level;

f Cost of providing trichiasis surgery at 80% coverage level;

g DALYS per year averted with trichiasis surgery provided at 80% coverage level.

Only one study reported global costs of VI comprehensively by including direct costs, productivity losses (morbidity- and mortality-related), informal care costs and intangible costs measured with DALYs.^32^ This study estimated that the total global cost in 2010 was $3,121 billion ppp, 85% of which was due to direct medical costs, in addition to 117.7 million DALYs (Table 5). Productivity losses were only estimated for high-income countries.^32^

Other global reports reported morbidity-related productivity losses. Productivity losses were estimated by several approaches including the use of disability weights for blindness and VI,^33^, ^34^, ^35^, ^36^ or for presbyopia,^37^ or simply assuming that people with VI and blindness have less chance of being employed^38^, ^39^, ^40^ and those who work received a lower wage.^39^ Reports ranged from $0.78 billion ppp for trachoma^41^ to $381 billion ppp for VI^39^ (Table 5).

#### Average cost estimates *general VI studies*

Average cost estimates *General VI Studies:* Most studies reporting average cost estimates restricted cost reporting to direct medical costs (35/41; 85%) (Tables 6 and 7). Only three studies reported annual informal care costs or productivity losses estimates in two high-income countries: Australia and Portugal. Cost estimates for informal care were similar between these two countries (<$1,000 ppp).^42^^,^^43^ Productivity loss estimates in Portugal were nearly ten times higher than informal care costs estimates, for the same population and year^44^ (Table 6). Average direct costs for VI and/or blindness were reported for the United Kingdom, the United States, China and South Korea as shown in Table 6. Cost estimates varied over time and between and within countries. In the United Kingdom, cost estimates of blindness in 2012 increased 2.5 times when compared to estimates in 1998.^45^^,^^46^ Higher estimates within the United Kingdom, were also found in studies assuming a broader perspective.^47^ Cost estimates for blindness in other countries varied between $3,799 ppp in South Korea^48^ and $5,946 in the United States^49^ (Table 6). The studies that reported age and/or sex-specific analysis showed that direct costs for blindness were higher in women than in men^46^ and rise consistently with increasing age.^46^^,^^48^^,^^49^

**Table 6:** Average annual cost estimates per person with vision impairment from any cause. Costs are in 2018 USD purchasing power parity.

Country	Reference	Cause	Treatment	Year of cost data	Sample size	Unit of Observation	Perspective of analysis	In 2018 USD ppp						Quality appraisal score
								Direct Costs						
								Medical (1)	Non- medical (2)	Total (1) + (2)	Productivity Losses	Informal Care costs		
**High-income Asia Pacific**														
South Korea	^48^	All causes a	Unspecified	2011	1810	Patient	Health System	3799	–	–	–	–		8
**Australasia**														
Australia	^104^^,^^105^	All causes	Unspecified	2003	150	Patient	Patient	217	598	815	–	917		9;8.5
Australia	^43^	All causes	Unspecified	2003	114	Patient	Patient	–	–	–	–	963		4.5
**Western Europe**														
Portugal	^42^	All causes	Unspecified	2014	546	Patient	Societal	–	–	–	–	848		8
Portugal	^44^	All causes	Unspecified	2014	546	Patient	Societal	–	–	–	10,124	–		8
United Kingdom	^46^	All causes	Unspecified	2012	3589	Patient	Health System	–	–	4971	–	–		9
United Kingdom	^45^	All causes	Unspecified	1997/98	3488	Patient	Health System	–	–	1841	–	–		9
United Kingdom	^47^	All causes	Unspecified	2000	N.R	Patient	Governmental	–	–	1st year: 131342nd year: 5675	–	–		8.5
Netherlands	^106^	All causes	Unspecified	2015	152	Patient	Societal	–	–	523 b	–	–		9
**High-income North America**														
United States	^49^	All causes	Unspecified	2004	10,796	Patient	Patient	1st year: 5946 2nd year: 12,808	–	–	–	–		8
**East Asia**														
China	^107^	All causes	Unspecified	2015	302	Patient	Patient	5181	2194	7374	–	–		9

USD – United States Dollars ($); ppp- purchasing power parity;.

a General VI study – all causes of VI;.

b Average costs for productivity losses and informal care costs reported together were reported to be $120 ppp.

**Table 7:** Average cost estimates for cataract treatment. Costs are in 2018 USD purchasing power parity.

Country	Reference	Cause	Treatment	Year of cost data	Sample size	Unit of Observation	Perspective of analysis	in 2018 USD ppp					
								Direct costs			Productivity Losses	Informal Care costs	Quality appraisal score
								Medical (1)	Non- medical (2)	Total (1) + (2)			
**High-income Asia Pacific**													
Japan	^56^	Cataract	Unspecified	2009	549	Episode a	Health System	3654 b	–	–	–	–	9
**Western Europe**													
United Kingdom	^108^	Cataract	Unspecified	1998/99	399	Patient	Patient	–	153;231 c	–	–	–	6.5
United Kingdom	^109^	Cataract	Phaco/ECCE	N.R	476	Surgery	Health System	–	–	725; 741 d	–	–	9.5
France	^55^	Cataract	Unspecified	2001	250	Episode	Health System	2690	–	–	–	–	8
France	^110^	Cataract	Unspecified	2011	125	Episode	Health System	465; 744 e	–	–	–	–	7
Sweden	^111^	Cataract	Unspecified	1998	565	Episode	Health System	–	787	–	–	–	7.5
9 EU countries	^112^	Cataract	Unspecified	2005	N.R	Patient	Health System	–	268 to 1673 f	–	–	–	8.5
**High Income North America**													
United States	^113^	Cataract	Unspecified	2012	N.R	Surgery	Health System and Societal	2934	–	–	–	–	9.5
United States	^54^	Cataract	Unspecified	2009	27	Episode	Payer	9615; 12,311 g	–	–	–	–	7
United States	^114^	Cataract	Unspecified	N.R	68,866	Episode	Payer	1087					3.5
United States	^115^	Cataract	Congenital Cataract surgery	2013	114	Patient	Payer	–	–	36,352; 38,353^h^	–	–	7.5
United States	^116^	Cataract	Unspecified	2004	137,039	Episode	Payer	–	–	3029	–	–	7.5
Canada	^117^	Cataract	Unspecified	2003	44	Episode	Hospital	1043 to 1542 g	–	–	–	–	8.5
**South Asia**													
India	^118^	Cataract	Congenital Cataract	2010	N.R	Episode	Hospital	–	140 to 547	–	–	–	8.5
India	^53^	Cataract	Phaco/ECCE/MSICS	2000	N.R	Surgery	Societal	–	–	23;24;36 i	–	–	8.5
India	^52^	Cataract	ECCE	1997	5025	Surgery	Societal	–	–	68 to 24	–	–	6.5
**Southeast Asia**													
Malaysia	^119^	Cataract	Phaco/ECCE	2000	247	Surgery	Health System	–	–	1288;1598 i	–	–	9.5
**East Asia**													
China	^120^	Cataract	Phaco	2000	1189	Surgery	Hospital	–	–	617 to 1488	–	–	8.5
**Eastern sub-Saharan Africa**													
Zambia	^50^	Cataract	ECCE	2010	40	Episode	Hospital and Patient	–	–	77	–	–	8
Kenya	^121^	Cataract	Paediatric cataract surgery	N.R	96	Patient	Hospital	303;380^g^	–	–	–	–	4
**Western sub-Saharan Africa**													
Nigeria	^51^	Cataract	Unspecified	N.R	104	Patient	Patient	–	–	54 j	–	–	8
**Tropical Latin America**													
Brazil	^122^	Cataract	ECCE	2001	1025	Surgery	Health System	786	–	–	–	–	6.5
Brazil	^123^	Cataract	Phaco/ECCE	N.R	205	Surgery	Health System	239; 349 i	–	–	–	–	6.5
Brazil	^124^	Cataract	Phaco	2000	58	Surgery	Health System	344	–	–	–	–	9

USD – United States Dollars ($); ppp- purchasing power parity; N.R – Not reported; Phaco, phacoemulsification surgery; ECCE, extracapsular cataract extraction; MSICS, manual small incision cataract surgery.

a Cataract surgery episode includes all costs involved in the pre-, intra and post-operative period (including out-patient attendance, post-operative attendance medication etc.) whereas ‘surgery` is just the surgical activity;

b Average cost for surgery in one eye;

c Average cost in two different hospitals: a district hospital and a community hospital (lowest value);

d Average costs for phaco (lowest value) and ECCE (highest value);

e Average costs for cataract surgery in outpatient settings (lowest value) and in inpatient settings (highest value);

f This study reported the cost of providing cataract surgery in nine countries in Europe: Denmark, England, France, Germany, Hungary, Italy and The Netherlands. Average costs varied considerably by country ranging from $286 ppp in Poland and $1673 ppp in Italy;

g Average cost for simultaneous bilateral surgery and sequential bilateral surgery (higher value);

h 5 year treatment cost: surgery and contact lenses (lowest value), surgery and intraocular lenses (highest value);

i Average costs for ECCE (lowest value), phaco (highest value);

j Average direct costs for men ($59 ppp highest value), women ($49 ppp lowest value), both sexes $54 ppp.

*Cataract:* Cost of cataract treatment varied considerably between countries and by type of surgery. The lowest estimates (<$150 ppp) were for extracapsular cataract extraction in lower-middle income countries including India, Nigeria and Zambia^50^, ^51^, ^52^, ^53^ (Table 7). The highest estimates per episode (>$2,500 ppp) were found in France, the United States and Japan.^54^, ^55^, ^56^

*Refractive error:* The cost of the provision of spectacles to correct refractive error was reported in three studies; costs ranged from $12 ppp in India^57^ to $201 ppp in a study undertaken in five European countries^58^ (Table 8).

**Table 8:** Average cost estimates for refractive error treatment. Costs are in 2018 USD purchasing power parity.

Country	Reference	Cause	Treatment	Year of cost data	Sample size	Unit of Observation	Perspective of analysis	in 2018 USD ppp					Quality appraisal score
								Direct Costs			Productivity Losses	Informal Care costs	
								Medical (1)	Non- medical (2)	Total (1) + (2)			
**Western Europe**													
Spain	^125^	Refractive Error	Unspecified	2014	48	Episode	Health System and Patient	569	30	599; 3577 a	**–**	**–**	8
5 EU countries	^58^	Refractive Error	Spectacles	N.R	4157	Spectacles	Societal	**–**	201 b	**–**	**–**	**–**	6
**South Asia**													
India	^57^	Refractive Error	Spectacles	2016	390	Episode	Health System	**–**	–	12;48 c	**–**	**–**	9.5
**Eastern sub-Saharan Africa**													
Zambia	^50^	Refractive Error	Spectacles	2010	43	Episode	Hospital and Patient	**–**	–	135	**–**	**–**	8

USD – United States Dollars ($); ppp- purchasing power parity; EU – European; N.R – Not reported.

a Average cost for treating high myopia (lowest value) and myopic choroidal neovascularisation (highest value);

b Average cost for 5 European countries: France, Germany, Italy, Spain, and the United Kingdom.

c Ready-made spectacles (lowest value $12 ppp); Custom made spectacles (highest value$ 48 ppp).

*Diabetic retinopathy:* Costs for treatment over a 5-year period were reported in the United States for diabetic retinopathy treated with anti-VEGF and estimated to be $40,825.^59^

*Glaucoma:* Among the 16 studies reporting costs for glaucoma, annual treatment costs varied between $878 ppp in Nigeria for surgical treatment^60^ and $5,272 ppp reported for four European countries for a much wider number of cost items such as rehabilitation care and home care costs^61^ (Table 9). Another study in the United States reported average 5-year costs for three glaucoma treatment strategies: medical treatment, trabeculectomy and tube insertion. Costs ranged from $6,707 for medical treatment to $10,949 for tube insertion.^62^

**Table 9:** Average cost estimate for glaucoma and diabetic retinopathy treatment. Costs are in 2018 USD purchasing power parity.

Country	Reference	Cause	Treatment	Sample size	Year of cost data	Unit of Observation	Perspective of analysis	in 2018 USD ppp					Quality appraisal score
								Direct costs			Productivity Losses	Informal Care costs	
								Medical (1)	Non- medical (2)	Total (1) + (2)			
**Western Europe**													
Finland	^126^	Glaucoma	All types a	168	2006	Patient	Health System	1354	**–**	**–**	**–**	**–**	9
4 EU countries b	^61^	Glaucoma	Unspecified	162	2005	Patient	Health System and Societal	1238	4034	5272	**–**	**–**	8
**High Income North America**													
United States	^127^	Glaucoma	All types a	81	N.R	Patient	Payer	–	**–**	1476; 2664 c	**–**	**–**	8
United States	^62^	Glaucoma	Surgical	N.R	2013	Episode	Health System	8555 d; 6707; 10,949	**–**	**–**	**–**	**-**	10
United States	^59^	Diabetic Retinopathy	Anti-VEGF	213	2018	Episode	Health System	40,825 e	**–**	**–**	**–**	**–**	8
United States	^128^	Diabetic Retinopathy	Unspecified	1441	2012	Patient	Payer	17,280 f	**–**	**–**	2210	**–**	5.5
**Western sub-Saharan Africa**													
Nigeria	^60^	Glaucoma	Surgical	120	2006	Patient	Patient and Governmental	878	**–**	**–**	–	**–**	8
**Tropical Latin America**													
Brazil	^129^	Glaucoma	Surgical	227	2010	Surgery	Health System	351;415;448^g^	**–**	**–**	–	**–**	7

USD – United States Dollars ($); ppp- purchasing power parity; EU – European; N.R – Not reported.

a It includes surgical, laser and medical treatment available in the country.

b France, Denmark, Germany and the United Kingdom.

c Average cost for second year of treatment (lowest value) and first year of treatment (highest costs);.

d Average cost of trabeculectomy treatment over 5 years ($8555 ppp), mean cost for medical treatment in the same time period was reported as$ 6707 ppp and for tube insertion as $10,949 ppp;.

e Average cost for a 5 year period for patients with proliferative diabetic retinopathy and center involved diabetic macular edema treated with ranibizumab.

f Average cost for diabetic retinopathy Non- drivers cohort. Commercial driver cohort data was not reported since it included exclusively persons for whom good vision is required to maintain employment.

g Average direct costs of non-penetrating deep sclerectomy by glaucoma severity level: early / moderate/ severe.

*AMD:* Studies that estimated costs of AMD reported costs for medical treatment, laser treatment and anti-VEGF treatment (Table 10). In general, anti-VEGF treatment studies were more recent and reported higher costs than any other AMD treatment strategy. Anti-VEGF treatment costs were estimated for several countries (Greece, United Kingdom, Switzerland, United States, Australia, South Korea and Turkey) and in several treatment periods and treatment regimens. For example, a report in the United Kingdom showed that, using bevacizumab in a discontinuous regimen ($4,824 ppp) was one sixth the cost of using ranibizumab in a continuous regimen ($29,872 ppp).^63^ The average annual cost of ranibizumab therapy also varied considerably between countries.^64^^,^^65^ Average costs ranged from $3,354 ppp in South Korea to $18,756 in the United States.^66^^,^^67^

**Table 10:** Average cost estimate for treatment of AMD, corneal opacity and trachoma. Costs are in 2018 USD purchasing power parity.

Country	Reference	Cause	Treatment	Year of cost data	Sample size	Unit of Observation	Perspective of analysis	In 2018 USD ppp						Quality appraisal score
								Direct costs			Productivity Losses	Informal Care costs		
								Medical (1)	Non- medical (2)	Total (1) + (2)				
**High-income Asia Pacific**														
Japan	^130^	AMD	Anti- VEGF	2017	71	Patient	Societal	–	–	–	–		778; 1512 a	7.5
Japan	^131^	AMD	Anti- VEGF	2013	3058	Patient	Payer	14,888 b	–	–	–		–	5.5
Korea	^66^	AMD	Unspecified	2014	7119	Episode	Payer	–	–	3354	–		–	8.5
**Australasia**														
Australia	^132^	AMD	Unspecified	N.R	103	Episode	Patient	1943	543	2486	982		2198	8.5
**Western Europe**														
3 EU countries	^133^	AMD	Unspecified	2004	360	Patient	Societal	3632	1608	5240 to 7524 c	–		–	8.5
France	^134^	AMD	Unspecified	2000	105	Patient	Payer	2934	2827	5762	–		1410	9
Germany	^135^	AMD	Unspecified	N.A	150	Patient	Caregiver	181	547	787	–		–	6.5
Greece	^136^	AMD	Anti- VEGF	2011	N.R	Patient	Payer	52,404 d	–	–	–		–	8.5
Ireland	^137^	AMD	Photodynamic therapy	2006	211	Patient	Societal	3377	536	3913	1950		1496	9
Italy	^138^	AMD	Laser	1999	476	Patient	Societal	767	–	–	–		–	8.5
Switzerland	^64^	AMD	Anti- VEGF	2014	3058	Episode	Payer	–	–	7747; 9424 e	–		–	5.5
Switzerland	^65^	AMD	Anti- VEGF	2016	361	Patient	Payer	10,692;12,456 f	–	–	–		–	8
United Kingdom	^63^	AMD	Anti- VEGF	2011	610	Patient	Health System	4824;29,871 g	–	–	–		–	9
United Kingdom	^139^	AMD	Photodynamic therapy	2007	4566; 1834^g^	Patient	Health System and Societal	–	–	2209;7911 h	–		–	7
**High Income North America**														
Canada	^140^	AMD	Photodynamic therapy	2005	166	Patient	Societal	5985	2498	8433	–		–	9
United States	^7^	AMD	Medical	1995–99	6290	Patient	Payer	2473 years up to $2710 for those aged 75- 79 years and then decreased (80-84 years, $2556; 85 years, $ 1800)"?>^i^	–	–	–		–	7.5
United States	^67^	AMD	Anti- VEGF	2009	N.R	Patient	Payer	16,261;18,756 j	–	–	–		–	4
United States	^141^	AMD	Anti- VEGF	2009	92	Patient	Payer	62,985	–	–	–		–	6.5
**Central Europe**														
Czech Republic	^142^	AMD	Anti- VEGF	2012	763	Patient	Hospital	9592	–	–	–		–	7.5
**North Africa, Middle East**														
Turkey	^143^	AMD	Anti- VEGF	2016	175	Patient	Payer	2657;5059^j^	–	–	–		–	6.5
**South Asia**														
India	^68^	Corneal Opacity	Medical	2004	498	Episode	Patient	112	4	116	39		–	4
**Western sub-Saharan Africa**														
Gambia	^69^	Trachoma	Trichiasis surgery	1998	120	Surgery	Societal	–	–	9	–		–	6.5

USD – United States Dollars ($); ppp- purchasing power parity; EU – European; N.R – Not reported; N.A – Not applicable.

a Average cost in the first year of treatment in two different regimens "as treat and extent regimen" ($778 ppp) and "as needed regimen" ($1512 ppp);

b Cost per 10 000 persons;

c Average cost for Italy ($5240 ppp), This study also reported cost for France (sum of direct costs $7524 ppp) and Germany (sum of direct costs $5920 ppp).

d Average cost for 10 years of ranibizumab treatment.

e Average cost for aflibecerpt ($7747 ppp); Average cost for ranibizumab $9424 ppp.

f Average monthly cost for aflibecerpt $1038 ppp, average monthly cost for ranibizumab $891 ppp.

g Lowest average reported value for discountinuous bevacizumab regimen, highest reported value for continuous ranibizumab regimen.

h Average cost of verteporfin photodynamic therapy (PDT): second year of treatment (lowest value) and first year of treatment (highest value) (including health and social service costs).

i Average costs for all patients; costs were stratified by age group, costs rose from $2362 for those aged 65-69 years up to $2710 for those aged 75- 79 years and then decreased (80-84 years, $2556; ≥85 years, $ 1800).

j Average cost for ranibizumab: year 2 (lowest value) and Year 1 (highest value).

*Corneal opacity and trachoma:* In India, one study reported, from a patient perspective, direct costs for treatment of corneal opacity of $116 ppp and associated productivity losses of $39 ppp.^68^ Trachoma surgery costs were estimated to be $9 ppp in The Gambia.^69^

#### Other studies

Other studies reported costs using different approaches. Eighteen studies reported costs by severity level, measured by visual acuity (e.g. moderate VI, severe VI, blindness) or various glaucoma and diabetic retinopathy classification systems. Different patterns and trends were observed across studies when average costs were split by visual acuity or by disease specific severity level although in two-thirds of studies higher vision loss or higher disease severity was associated with higher costs (supplementary Table 5 p 13–15). For glaucoma reported annual average costs ranged from $410 ppp in Canada (direct medical costs reported for glaucoma patients with moderate VI)^70^ to $32,903 in the United States (direct medical and non-medical costs for glaucoma patients with VI).^71^ A similar wide range of costs was found for AMD but this time the lowest cost report was found in a study in Thailand.^72^ Inclusion of home care, informal care or institutional care costs increased total costs considerably, regardless of the cause of VI. None of the studies reporting direct non-medical costs related to home care or institutional costs reported costs below $14,000 (maximum report of $96,588 for glaucoma patients with very severe VI^73^).

Comparisons between ophthalmic and non- ophthalmic costs were reported in six studies^74^, ^75^, ^76^, ^77^, ^78^, ^79^ (supplementary Table 6 p 16). Direct ophthalmic costs accounted for 7% to 70% of total cost. Non-ophthalmic costs included a wide variety of costs such as costs related to falls and fractures, depression and anxiety treatment and primary care visits. The annual direct ophthalmic and non-ophthalmic treatment costs per AMD patient ranged between $7,721 ppp in the United Kingdom^77^ and $38,665 in the United States.^79^

#### Search update

Our search updated in January 2022 identified 487 potential studies. We reviewed the full text of 53 of these and ultimately identified 14 eligible studies. The main characteristics of these studies are listed in supplementary Table 7 (supplementary Appendix p17). These additional studies do not change the conclusions of our review i.e. they were predominantly undertaken in high-income countries (*n* = 10, 71%) and tended to take a prevalence-based, bottom-up approach to report direct costs.

## Discussion

To our knowledge, this systematic review is the first to comprehensively summarise findings and methodological considerations of studies estimating the costs associated with VI and its major causes across all world regions. It is also the first systematic review to document the costs associated with anti-VEGF treatment for eye conditions. We identified 38 studies reporting data for VI and 100 studies reporting data for one or more of seven major causes of VI. Two-thirds of studies reported data from high-income countries, highlighting the need for more studies that estimate the economic burden of VI in low- and middle-income countries, where 80% of the global population and 90% of people with VI live.^2^

In addition, we found considerable variation and limitations in the VI cost literature. The methods used, and the results reported were, in general, not standardised. The costs reported were not comprehensive and inadequate sensitivity analysis was performed. This widely recognized variation in methods and reporting^80^^,^^81^ reduces the generalizability and comparability of studies and compromises the usefulness of cost studies to inform priority setting decisions.^82^^,^^83^

We found that treatment costs for all causes of VI varied considerably between and within countries, reflecting variation in methodological and reporting approaches, and differences in health care systems including therapeutic options and regimens, organizational systems, clinical pathways and resources. Treatment options for refractive error, trachoma and cataract tended to be less expensive than those for AMD, diabetic retinopathy and glaucoma. The cost of treating cataract has tended to reduce over time in low-, middle- and high-income countries (Table 8). In contrast, the introduction of anti-VEGF treatment for AMD has increased costs of AMD treatment, though the range of anti-VEGF medication regimens resulted in many different costs estimates (Table 10).

In this systematic review we found a lack of clarity and uniformity regarding the cost items included in the four major cost components: direct costs, productivity costs, informal care costs and intangible costs. Many studies did not disaggregate and disclose the type and number of cost components included in the estimated cost which made it impossible to identify the main cost drivers. Moreover, the majority of studies (53%), even those classified as adopting a societal perspective, included only a limited number of cost components, which contributed to a lack of comprehensiveness in the cost estimates generated. Even within direct medical costs reported, we found significant variation, with some studies including only physician visits and medication, and excluding important items such as medical examination and rehabilitation care. The type and extent of non-ophthalmic costs included, such as those related to comorbidities (e.g. depression and anxiety) or to sequelae of VI (e.g. falls and fractures) meant the proportion of direct costs attributed to ophthalmic versus non-ophthalmic costs varied widely (7% to 70% of total costs) and further highlights the lack of uniformity and generalizability of costs estimates.^74^, ^75^, ^76^, ^77^, ^78^, ^79^

The widespread use of different assumptions and models to estimate productivity losses,^33^, ^34^, ^35^, ^36^, ^37^, ^38^^,^^40^ also emphasises the lack of more reliable and up-to-date data sources across a wide range of regions and a lack of consistency in the application of analytic methods. For example, global productivity losses for trachoma varied between $0.78 billion ppp and $7.10 billion ppp due to differences in costing methods and the trachoma definition used to identify prevalent cases (Table 5).^36^^,^^41^

The inadequate characterization of uncertainty in 70% of studies also constitutes an important limitation of the current VI literature. Such analysis, generally recommended in economic evaluation textbooks and guidelines, is an integral component of any robust and transparent economic analysis as it aids understanding and assessment of the limitations of studies and also identifies the key variables for which more precise measurement is needed to improve future studies.^84^

Our review must be considered in the context of several limitations. First, our inclusive approach to capture any cost estimate for VI or its major causes from any perspective contributed to the substantial methodological heterogeneity observed across included studies. Estimating societal costs of a health problem is different from estimating the incremental cost per patient of a specific intervention and implies different methodological approaches.^20^ Second, we restricted our search to papers published from January 2000 onwards and therefore we may have reduced the number of included studies. We believe that restricting inclusion for studies published no more than 20 years ago could increase generalisability to current and future years due to changes in standard of care and research methods.^19^ Moreover, to minimise the risk of missing relevant studies we searched the three most commonly used sources: Medline, CRD database (which includes NHS EED and DARE databases) and the HTA database,^19^^,^^85^ and included an information specialist (IG) to help with the search strategy.^86^ Risk of bias in literature selection was also minimised by performing each step of the selection process, independently and in duplicate.^19^

To improve future costs estimates, we recommend relevant stakeholders develop a “reference case” for eye health - a reference document of costing methods based on well-defined principles that can support better decision through standards for planning, conducting and reporting and enable more robust and consistent decisions over time.^87^^,^^88^ This reference case also includes a list of standardised unit cost for eye care interventions and services, as demonstrated by a similar process followed by the Global Health Cost Consortium for tuberculosis.^89^

There are a couple of guidelines and recommendations in the field of economic evaluation, namely for cost effectiveness analysis in all fields^83^ and in the eye care field,^90^ nonetheless, these guidelines provide a very broad spectrum of recommendations mainly for reporting^83^ providing less details about the methods and process behind cost estimation. A reference case would go beyond these existing recommendations to provide a framework that allows institutions or individuals estimating costs to structure their choices around study design and methods, data sources availability and to consider how their costing methods influence the quality and introduce limitations to their estimates. Once adopted, this reference case would serve to improve the quality of cost estimates by ensuring consistency, coherency, transparency of methods, assumptions and reporting.^89^ These objectives might be implemented by a “comply or justify” approach that allows analysts to adapt their approach to specific contexts and requirements but requires that their judgements about methodological choices are made explicitly and transparently.^87^

Regular cost reports that ensure comparability and facilitate trend analysis over time, between settings and between other conditions will be worthwhile once methods and reporting have been standardised. For example, multi-country cost of illness studies could be used to describe or to predict the extent of changes in different settings or to analyse distribution of costs over time. We also need to develop processes to identify and determine the main cost drivers for future benchmarking to improve quality of care, as well as productivity and efficiency of resources and funding allocation. These studies are needed everywhere but particularly in the eight GBD regions (Central Asia, Eastern Europe, Southern Latin America, Caribbean, Andean Latin America, Oceania, Central sub-Saharan Africa and Southern sub-Saharan Africa) where no estimates were identified with this review. Standardisation of methods and cost reporting will also allow the development of a wider range of economic studies such as cost effectiveness analysis, budgetary impact and feasibility analysis which are very useful to better inform countries in making decisions on the delivery of eye care services and evaluating the socioeconomic impact of VI worldwide. Future research should aim to fill these gaps, adding new data sources and adopting new standards for methods and cost reporting.

Robust studies that report costs of a health condition contribute to an understanding of the economic burden of the condition on the overall population, which in turn informs planning and financing decisions and future economic evaluations.^16^^,^^20^^,^^91^ Our review has highlighted that the existing literature on costs of VI in many countries is insufficient to be easily used for these purposes.

To achieve a more complete picture of the global cost of VI, more studies must be conducted in all the seven leading causes of VI and in low- and middle-income countries and studies everywhere must be done more regularly with standardised methods and reporting. To help the development and compliance of standardised methods we recommend the development and adoption of a reference case to guide future cost estimates of eye health (including VI) and eye health interventions and services.

## Contributors

APM, JC, JR and MJB conceived the idea for the review and with all other authors designed the protocol. IG constructed the search. APM, JR, JZ and ThB selected the studies and extracted the relevant data. APM, ThB, IJ, AN and MJ appraised studies. APM, JR, JC and MJB wrote the original draft and JZ, ThB, KF, IJ, HF, AB, HT, RB, IG, AN, MJ, TaB, SR, PK critically revised and edited successive drafts of the paper. All authors gave final approval of the version to be submitted.

### Data sharing statement

The protocol for this study has been published previously. As this is a review of the literature, there are no new original data in this article to be shared.

### Funding

We acknowledge funding support for *The Lancet Global Health Commission on Global Eye Health* from The Queen Elizabeth Diamond Jubilee Trust, The Wellcome Trust, Moorfields Eye Charity (GR001061), Sightsavers, The Fred Hollows Foundation, The SEVA Foundation, The British Council for the Prevention of Blindness, and Christian Blind Mission. MJB is supported by the Wellcome Trust (207472/Z/17/Z).

## Declaration of interests

MJB reports grants, in support of the work for *The Lancet Global Health Commission on Global Eye Health*, from The Queen Elizabeth Diamond Jubilee Trust, The Wellcome Trust, Moorfields Eye Charity (GR001061), Sightsavers, The Fred Hollows Foundation, The SEVA Foundation, The British Council for the Prevention of Blindness, and Christian Blind Mission. MJB is supported by the Wellcome Trust (207472/Z/17/Z). PK reports grants from Wellcome Trust, The Helen Hamlyn Trust, UCL Technology Fund, Moorfields Eye Charity, The Jules Thorn Charitable Trust, Fight for Sight, National Institute for Health Research, Apollo Therapeutics Fund, Medical Research Council, personal fees from Aerie, Alcon, CMER Hospital Group, Genetech, Glaukos, ISA RNA Therapeutics GmBH, Novartis, Santen, and Thea outside of the submitted work; having a patent pending for Biochannel Device, PK Eye Model, Moorfields UCL MIO Muller stem cells; participation on advisory boards for (Novartis, DrugTech, Santen, CRICK Institute, Decisions in health Care to Introduce or Diffuse innovations using Evidence); being a board member of Moorfields Eye Hospital, Ophthalmology Foundation Board, UCL Partners Academic Health Science Centre, UCLP Informatics Board; being a member of Clinical Research Coalition Group, AHSC Planning & Performance Executive, UCL IOO & Moorfields Joint Campaign and Steering Group, Faculty of Brain Sciences Heads of Research Departments Board, Joint Research Governance Committee, AHSC and AHSN Programme Directors Forum, Moorfields Eye Hospital and UCL Institute of Ophthalmology, R&D Advisory Group of Central & East London Local Clinical Research Network, Joint Research Strategy Committee, Moorfields Eye Hospital Institute of Ophthalmology, Research Management Committee, Moorfields Eye Hospital; being a director of NIHR Biomedical Research Centre for Ophthalmology; being on the grant panel of Moorfields Eye Charity, ICO-Allergan Research Fellowship Program, Alcon Research Institute Scientific Select Committee, Ruskell Medal Reviewer Committee, Worshipful Company of Spectacle; being a founder of and stockholding for Radiance Therapeutics and Optceutics; being a co-founder of Lumemed Let and being a private clinician at Moorfields Private.

## Appendix Supplementary materials

Image, application 1

Image, application 2
