# Supplementary material for: The economics of vision impairment and its leading causes: A systematic review
Source: eClinicalMedicine. 2022 Mar 22;46:101354. doi: 10.1016/j.eclinm.2022.101354 (PMC8943414; doi:10.1016/j.eclinm.2022.101354)
Supplement: Supplementary file 2 [file mmc2.docx]

## 1. Search strategies

**MEDLINE (Ovid)**

1. "Global Burden of Disease"/

2. "costs and cost analysis"/

3. cost-benefit analysis/

4. "cost of illness"/

5. health care costs/

6. "Health Services Needs and Demand"/ec, sn [Economics, Statistics & Numerical Data]

7. Health Care Surveys/ec, sn [Economics, Statistics & Numerical Data]

8. Health Expenditures/ec, sn [Economics, Statistics & Numerical Data]

9. Health Resources/ec, sn [Economics, Statistics & Numerical Data]

10. Global Health/ec, sn [Economics, Statistics & Numerical Data]

11. ((global or economic) adj2 burden).tw.

12. ((cost or costs) adj2 (benefit or analysis or illness or direct or indirect or Intangible)).tw.

13. Efficiency/

14. Absenteeism/

15. Presenteeism/

16. productivity.tw.

17. "Severity of Illness Index"/ec [Economics]

18. Employment/ec [Economics]

19. Sick Leave/ec, sn [Economics, Statistics & Numerical Data]

20. (absenteeism or presenteeism or productivity).tw.

21. Caregivers/ec, sn [Economics, Statistics & Numerical Data]

22. or/1-21

23. exp eye diseases/

24. exp vision disorders/

25. ((vision or visual$) adj2 (impair$ or loss or disorder)).tw.

26. (cataract$ or glaucoma or macula$ degeneration).tw.

27. (diabetic retinopathy or refractive error$ or trachoma or corneal opacity).tw.

28. or/23-27

29. 22 and 28

30. limit 29 to yr="2000 -Current"

**CRD database**

The CRD database will be searched using the following MeSH terms:

MeSH DESCRIPTOR Eye Diseases EXPLODE ALL TREES

MeSH DESCRIPTOR Vision Disorders EXPLODE ALL TREES

The protocol for this systematic review was registered on Open Science Framework (<https://osf.io/9au3w> - doi10.17605/OSF.IO/6F8VM) and published ^1^.

## 2. Methodological complementary information

Criteria for assigning epidemiological approach, resource quantification method, perspective of analysis and study design

Studies could be classified as following two different epidemiological approaches: prevalence or incidence-based approaches. Prevalence-based studies estimate costs associated with prevalent cases over a given period of time, (usually 1 year), while incident-based studies estimate costs accrued over a lifetime following the onset of the illness or loss of health state. Resource consumption methods are usually categorized into top-down (“population based”) or bottom-up (“person-based”). Top-down method uses aggregate expenditures by cost component collected in published expenditures, administrative databases (eg. provider databases, claims/billing databases), and population surveys among others. Bottom-up method assigns costs to individuals with a specific disease or condition collected in, but not limited to patients diaries, patient clinical records and specially design questionnaires.

Studies were also classified in several perspectives, including societal, governmental, healthcare system, payer, healthcare provider and patient. Whenever not stated perspective of analysis was defined in accordance with the type of reported costs (e.g studies reporting productivity loss costs due to VI and its major causes incurred by the patient himself or the caregivers either alone or in conjunction with other costs categories were considered to follow a societal perspective) and/or in accordance to the entity that is intended to be informed with the study usually the entity responsible for providing or paying for the reported costs.

We classified studies as a cost of illness study if they identified, measured and described all the costs associated with a condition (VI) or a disease (7 major causes of VI), as cost analysis if they compared costs between different groups of patients, different types of treatments, different settings and different stages of diseases and as cost effectiveness analysis if studies compared alternative treatments in terms of both the costs and consequences.

Cost categories definition

Due to heterogeneity in the cost data, studies were stratified by the four major costs components categories (i.e. direct costs, productivity losses, informal care and intangible costs). Direct costs were further divided into direct medical costs and direct non-medical costs. Direct medical costs were considered costs associated with inpatient care, outpatient care, medical prescriptions, medical examinations or rehabilitation care. Direct nonmedical costs were considered costs attributed to home care, institutional care, vision aids and devices, home modifications, transport costs or government programs for people who have vision impairment or blindness.

We adopted the term productivity loss costs instead of indirect costs, as formerly used, to distinguish clearly the economic value of production forgone associated with loss or reduction of employment (productivity cost concept) from the concept of indirect costs used in the accounting system to designate overhead costs. We split productivity costs into morbidity-related productivity losses (absenteeism, presenteeism, reduced workforce participation etc) and mortality-related productivity losses (productivity losses due to premature mortality).

Informal care costs refers to monetary estimate of hours spent by caregivers.

Intangible costs refer to patient’s psychological pain, discomfort, anxiety and distress. Such costs are usually expressed in the form of quality-of-life measures.

Cost transformation details

To enhance the comparability of the data costs were inflated to 2018 values using a country-specific gross domestic product deflator, and then converted to USD purchasing power parities (PPP) to equalise the purchasing power of different currencies (not required when studies were undertaken in the United States and reported in USD).

Where the World Bank database did not report information for a study country or region identified in our review, we used national sources to transform cost data to the year 2018 and PPP (Quandl Inc, Toronto, Canada available at: https://www.quandl.com/). For European countries such as Hungary or Poland that have not adopted Euros, but for which reported costs were made in Euros, costs were inflated to 2018 using the World Bank database and then converted from Euros to local currency using exchange rates for 31 December 2018 published by the European Central Bank and from local currency to USD PPP using the World Bank database. More details are given in the protocol ^1^.

Cost categories recalculation

Due to a lack of standard denominations and classification on the four major cost components found in the included studies, whenever possible, cost categories were recalculated to increase comparability between studies. The following framework was used:

| 1. Direct Medical Costs |
| --- |
| 1.1 Inpatient care |
| 1.2 Outpatient care |
| 1.2.1 Hospital Outpatient care |
| 1.2.2 Other Outpatient care |
| 1.2.3 Medications |
| 1.2.4 Medical Exams |
| 1.2.5 Rehabilitation care (includes vision aids if prescribed by a health professional) |
| 2. Direct Non-Medical Costs |
| 2.1 Vision aids and devices |
| 2.2 Home care |
| 2.2 Institutional care |
| 2.3 Transportation, food and accommodation |
| 2.4 Disability benefits |
| 2.5 Adaption of living environment |
| 2.6 Others (e.g: education + job training or Investments costs or nutrition costs) |
| 3. Productivity losses |
| 3.1 Productivity losses - morbidity related |
| 3.1.1 Absenteeism |
| 3.1.2 Presenteeism |
| 3.1.3 Reduced employment participation |
| 3.1.4 Reduced wages |
| 3.1.5 worktime lost due to travel and waiting |
| 3.2 Productivity losses - mortality |
| 3.2.1 Premature mortality |
| 4. Informal care costs |
| 5. Intangible costs/loss of well-being measures |
| 5.1 Disability Adjusted Life Years (DALYs) |
| 5.2 Quality Adjusted Life Years (QALYs) |

## Supplementary table 1: Cost components and intangible costs/loss of well-being measures reported in each included study (see reference list page 18 to 23 for study ID)

| **Reference**  (List at end of annex) | **Direct Costs** | | **Productivity Losses** | | **Informal care costs** | **Intangible costs** | |
| --- | --- | --- | --- | --- | --- | --- | --- |
|  | **Medical** | **Non-Medical** | **Morbidity** | **Mortality** |  | **DALYs** | **QALYS** |
| ^2^ |  |  | x |  |  |  |  |
| ^3^ | x | x | x | x |  | x |  |
| ^4^ |  |  | x |  |  |  |  |
| ^5^ | x | x |  |  |  |  | x |
| ^6^ | x |  |  |  |  |  |  |
| ^7^ |  |  | x |  |  |  |  |
| ^8^ | x |  |  |  |  |  |  |
| ^9^ |  |  |  |  |  |  | x |
| ^10^ | x | x | x | x | x | x |  |
| ^11^ | x | x |  |  |  | x |  |
| ^12^ | x | x |  |  | x |  |  |
| ^13^ | x | x |  |  | x |  |  |
| ^14^ | x | x |  |  | x |  |  |
| ^15^ |  |  |  |  | x |  |  |
| ^16^ |  | x |  |  |  |  |  |
| ^17^ | x | x |  |  |  |  |  |
| ^18^ | x | x | x | x | x | x |  |
| ^19^ | x | x |  |  |  |  |  |
| ^20^ | x | x |  |  |  |  |  |
| ^21^ |  | x | x |  | x |  |  |
| ^22^ | x |  | x |  | x |  |  |
| ^23^ | x | x | x | x | x | x |  |
| ^24^ | x | x |  |  |  |  |  |
| ^25^ | x | x | x |  |  |  |  |
| ^26^ |  | x | x |  | x |  |  |
| ^27^ |  |  |  |  | x |  |  |
| ^28^ |  |  | x |  |  |  |  |
| ^29^ | x | x | x |  |  |  |  |
| ^30^ | x |  |  |  | x |  |  |
| ^31^ | x |  |  |  |  |  |  |
| ^32^ | x | x | x |  | x | x |  |
| ^33^ | x |  |  |  |  |  |  |
| ^34^ | x | x | x |  |  |  | x |
| ^35^ | x |  | x | x | x | x |  |
| ^36^ |  | x |  |  |  |  |  |
| ^37^ |  |  | x |  |  |  |  |
| ^38^ |  |  | x |  |  |  |  |
| ^39^ |  |  |  |  |  |  |  |
| ^40^ | x | x |  |  |  |  |  |
| ^41^ |  | x |  |  |  |  |  |
| ^42^ | x | x | x |  |  |  |  |
| ^43^ | x | x |  |  |  |  |  |
| ^44^ | x | x | x |  | x |  |  |
| ^45^ | x |  |  |  |  |  |  |
| ^46^ | x | x |  |  |  |  |  |
| ^47^ |  |  | x |  |  |  |  |
| ^48^ |  |  | x |  | x |  |  |
| ^49^ |  | x |  |  |  |  |  |
| ^50^ |  |  | x |  |  |  |  |
| ^51^ | x |  |  |  |  |  | x |
| ^52^ | x |  |  |  |  |  |  |
| ^53^ | x |  |  |  |  |  |  |
| ^54^ | x | x |  |  |  |  |  |
| ^55^ |  | x |  |  |  |  |  |
| ^56^ | x | x |  |  |  |  |  |
| ^57^ | x | x |  |  | x |  |  |
| ^58^ | x |  |  |  |  |  |  |
| ^59^ | x | x |  |  |  |  |  |
| ^60^ | x |  |  |  |  |  |  |
| ^61^ | x |  |  |  |  |  |  |
| ^62^ | x |  |  |  |  |  |  |
| ^63^ | x | x |  |  |  |  |  |
| ^64^ | x | x |  |  |  |  |  |
| ^65^ | x |  |  |  |  |  |  |
| ^66^ | x |  |  |  |  |  |  |
| ^67^ | x |  |  |  |  |  |  |
| ^68^ | x |  |  |  |  |  |  |
| ^69^ |  |  | x |  |  |  | x |
| ^70^ | x | x |  |  |  |  |  |
| ^71^ | x | x |  |  |  |  |  |
| ^72^ |  | x |  |  |  |  |  |
| ^73^ | x | x |  |  |  |  |  |
| ^74^ | x | x |  |  |  |  |  |
| ^75^ | x | x |  |  |  |  |  |
| ^76^ | x |  |  |  |  |  |  |
| ^77^ |  |  | x |  |  |  |  |
| ^78^ | x |  |  |  |  | x |  |
| ^79^ | x | x | x |  | x | x |  |
| ^80^ | x |  |  |  |  |  |  |
| ^81^ | x |  |  |  |  |  |  |
| ^82^ | x | x |  |  |  |  |  |
| ^83^ | x |  |  |  |  |  |  |
| ^84^ | x | x |  |  |  |  |  |
| ^85^ | x | x |  |  |  |  |  |
| ^86^ | x |  |  |  |  |  |  |
| ^87^ | x |  |  |  |  |  |  |
| ^88^ | x | x |  |  |  |  |  |
| ^89^ | x |  |  |  |  |  |  |
| ^90^ | x | x |  |  |  |  |  |
| ^91^ | x |  |  |  |  |  |  |
| ^92^ | x |  |  |  |  |  |  |
| ^93^ | x |  |  |  |  |  |  |
| ^94^ | x | x | x |  | x |  |  |
| ^95^ | x |  |  |  |  |  |  |
| ^96^ |  |  |  |  | x |  |  |
| ^97^ | x |  |  |  |  |  |  |
| ^98^ | x | x |  |  |  |  |  |
| ^99^ | x | x | x |  | x |  |  |
| ^100^ | x |  |  |  |  |  |  |
| ^101^ | x |  |  |  |  |  | x |
| ^102^ | x | x |  |  |  |  |  |
| ^103^ | x | x |  |  | x |  |  |
| ^104^ | x |  |  |  |  |  |  |
| ^105^ | x |  |  |  |  |  |  |
| ^106^ | x | x |  |  |  |  |  |
| ^107^ | x | x |  |  |  |  |  |
| ^108^ | x |  |  |  |  |  |  |
| ^109^ | x |  |  |  |  |  |  |
| ^110^ | x | x |  |  |  |  | x |
| ^111^ | x | x | x |  | X |  |  |
| ^112^ | x |  |  |  |  |  |  |
| ^113^ |  |  |  |  | x |  |  |
| ^114^ | x | x |  |  |  |  |  |
| ^115^ |  |  |  |  |  |  | X * |
| ^116^ | x | x |  |  |  |  |  |
| ^117^ | x | x | x |  |  |  |  |
| ^118^ | x |  |  |  |  |  |  |
| ^119^ | x |  |  |  |  |  |  |
| ^120^ | x |  |  |  |  |  |  |
| ^121^ | x |  |  |  |  |  |  |
| ^122^ | x | x |  |  | x |  |  |
| ^123^ |  |  |  |  | x |  |  |
| ^124^ | x | x |  |  |  |  |  |
| ^125^ | x |  |  |  |  |  |  |
| ^126^ | x | x |  |  | x |  |  |
| ^127^ |  |  |  |  | x |  |  |
| ^128^ | x | x | x |  | x | x |  |
| ^129^ | x | x | x |  |  |  |  |
| ^130^ | x |  |  |  |  |  |  |
| ^131^ | x |  |  |  |  |  | x |
| ^132^ | x |  | x |  |  |  |  |
| ^133^ | x | x |  |  |  |  |  |
| ^134^ | x | x |  |  |  |  |  |
| ^135^ | x | x |  |  |  |  |  |
| ^136^ | x |  |  |  |  | x |  |
| ^137^ |  |  | x |  |  |  |  |
| ^138^ |  |  | x |  | x |  |  |
| ^139^ |  |  | x |  |  |  |  |

## Supplementary table 2: Distribution of included studies by geographic region and condition

|  | **General VI studies (n=38)** | **Condition specific studies (n=100)** | | | | | | | **Total**  **(n=138)** |
| --- | --- | --- | --- | --- | --- | --- | --- | --- | --- |
|  |  | **URE (n=11)** | **Cataract (n=28)** | **Glaucoma (n=16)** | **AMD (n=33)** | **DR (n=6)** | **Corneal Opacity**  **(n=1)** | **Trachoma (n=5)** |  |
| **7 Super Regions + World** | | | | | | | | | |
| Central Europe, Eastern Europe, and Central Asia | 1 | 0 | 1 | 0 | 1 | 0 | 0 | 0 | 3 |
| High Income | 30 | 4 | 15 | 12 | 30 | 5 | 0 | 0 | 96 |
| Latin America and Caribbean | 1 | 0 | 3 | 2 | 1 | 0 | 0 | 0 | 7 |
| North Africa and Middle East | 0 | 1 | 0 | 0 | 1 | 0 | 0 | 0 | 2 |
| Southeast Asia, East Asia, and Oceania | 3 | 0 | 4 | 0 | 1 | 0 | 0 | 0 | 8 |
| South Asia | 2 | 1 | 4 | 1 | 0 | 1 | 1 | 0 | 10 |
| Sub-Sharan Africa | 1 | 1 | 4 | 1 | 0 | 0 | 0 | 1 | 7 |
| World | 5 | 4 | 1 | 0 | 0 | 0 | 0 | 4 | 14 |
| **Super Region total estimates** | **43**^a^ | **11** | **32** ^b^ | **16** | **34**^c^ | **6** | **1** | **5** | **147** ^d^ |
| **21 Regions + world** | | | | | | | | | |
| Central Asia | 0 | 0 | 0 | 0 | 0 | 0 | 0 | 0 | 0 |
| Central Europe | 1 | 0 | 1 | 0 | 1 | 0 | 0 | 0 | 3 |
| Eastern Europe | 0 | 0 | 0 | 0 | 0 | 0 | 0 | 0 | 0 |
| Asia Pacific | 4 | 1 | 1 | 0 | 5 | 0 | 0 | 0 | 11 |
| Australasia | 10 | 0 | 0 | 1 | 2 | 1 | 0 | 0 | 14 |
| Western Europe | 11 | 2 | 8 | 4 | 16 | 2 | 0 | 0 | 43 |
| Southern Latin America | 0 | 0 | 0 | 0 | 0 | 0 | 0 | 0 | 0 |
| North America | 7 | 1 | 6 | 7 | 12 | 2 | 0 | 0 | 35 |
| Caribbean | 0 | 0 | 0 | 0 | 0 | 0 | 0 | 0 | 0 |
| Andean Latin America | 0 | 0 | 0 | 0 | 0 | 0 | 0 | 0 | 0 |
| Central Latin America | 1 | 0 | 0 | 1 | 0 | 0 | 0 | 0 | 2 |
| Tropical Latin America | 1 | 0 | 3 | 1 | 1 | 0 | 0 | 0 | 6 |
| North Africa and Middle East | 0 | 1 | 0 | 0 | 1 | 0 | 0 | 0 | 2 |
| South Asia | 2 | 1 | 4 | 1 | 0 | 0 | 1 | 0 | 9 |
| East Asia | 2 | 0 | 1 | 0 | 0 | 0 | 0 | 0 | 3 |
| Southeast Asia | 1 | 0 | 3 | 0 | 1 | 1 | 0 | 0 | 6 |
| Oceania | 0 | 0 | 0 | 0 | 0 | 0 | 0 | 0 | 0 |
| Central sub-Saharan Africa | 0 | 0 | 0 | 0 | 0 | 0 | 0 | 0 | 0 |
| Eastern sub-Saharan Africa | 0 | 1 | 3 | 0 | 0 | 0 | 0 | 0 | 3 |
| Southern sub-Saharan Africa | 0 | 0 | 0 | 0 | 0 | 0 | 0 | 0 | 0 |
| Western sub-Saharan Africa | 1 | 0 | 1 | 1 | 0 | 0 | 0 | 1 | 4 |
| World | 5 | 4 | 1 | 0 | 0 | 0 | 0 | 4 | 14 |
| **Region total estimates** | **46 ^e^** | **11 ^f^** | **32** | **16** | **39 ^g^** | **6** | **1** | **5** | **155 ^h^** |

URE = Uncorrected Refractive Error; AMD = Age-related macular degeneration; DR = Diabetic Retinopathy

^a^ 2 studies report data in more than one GDB Super region (Chakravarthy et al ^2^= Central Europe, Eastern Europe, and Central Asia and High Income; Eckert al ^4^ = High Income, Latin America and Caribbean, Sub-Saharan Africa, South Asia, Southeast Asia East Asia and Oceania)

^b^ 2 studies report data in more than one GDB Super Region (Fatore et al ^54^ = Central Europe, Eastern Europe, and Central Asia and High Income; Polack et al ^77^ = Southeast Asia, East Asia, and Oceania, South Asia and Sub-Saharan Africa)

^c^ 1 study reports data in more than one GDB Super region (Varano et al ^127^ = High Income and Latin America and Caribbean)

^d^ 5 studies report data in more than one GDB Super region and 1 study reports data in more than one condition (Griffith et al ^46^ = Uncorrected Refractive Error and Cataract in Sub-Saharan Africa Super Region)

^e^ 2 studies report data in more than one GDB Region (Chakravarthy et al ^2^ = Central Europe and Western Europe; Eckert et al ^4^ = Asia Pacific, Western Sub-Saharan Africa, North America, Central Latin America, South Asia, Australasia and Southeast Asia)

^f^ 1 study reports data in more than one condition (Griffith et al ^46^= Uncorrected Refractive Error and Cataract in Sub-Saharan Africa Super Region) and 2 studies reports data in more than one GDB Region (Fattore et al ^54^ = Western Europe and Central Europe; Polack et al ^77^= Southeast Asia, South Asia and East Sub-Saharan Africa)

^g^ 3 studies report data in more than one GDB region (Varano et al ^127^ = Australasia, Tropical Latin America, North America, Western Europe, Asia Pacific; Cruess et al ^114^ = North America and Western Europe; Soubrane et al ^115^ = North America and Western Europe)

^h^ 5 studies report data in more than one GDB region (Fattore et al ^54^ = Western Europe and Central Europe; Polack et al ^77^ = Southeast Asia, South Asia and East Sub-Saharan Africa; Varano et al ^127^ = Australasia, Tropical Latin America, North America, Western Europe and Asia Pacific; Cruess et al ^114^= North America and Western Europe; Soubrane et al ^115^ = North America and Western Europe)

## Supplementary table 3: Summary of quality appraisal scores for each item of the British Medical Journal Checklist for economic submissions adapted for cost of illness studies

| **Question** | **Yes (1)** | **No (0)** | **Not applicable (n.a)** | **Partially (p)** | **Ideal Scores ^a^** |
| --- | --- | --- | --- | --- | --- |
| Was a clear definition of the illness given? | 97 | 20 | 0 | 21 | 138 |
| Were epidemiological sources carefully described? | 123 | 3 | 3 | 9 | 135 |
| Were direct/ indirect costs sufficiently disaggregated? | 78 | 34 | 6 | 20 | 132 |
| Were activity data sources appropriately described? | 113 | 10 | 1 | 14 | 137 |
| Were activity data sources carefully assessed? | 111 | 14 | 0 | 13 | 138 |
| Were the sources of all costs values analytically described? | 91 | 12 | 5 | 30 | 133 |
| Were unit cost appropriately valued? | 97 | 20 | 4 | 17 | 134 |
| Were the methods adopted carefully explained? | 108 | 3 | 0 | 27 | 138 |
| Were the major assumptions tested in a sensitivity analysis? | 46 | 90 | 1 | 1 | 137 |
| Was the presentation of study results consistent with the methodology of the study? | 106 | 2 | 0 | 30 | 138 |
| **Sum** | **970** | **208** | **20** | **182** | **1360** |

^a^ An ideal score corresponded to the total number of studies with an applicable response for each item (i.e. the studies for which the item was not applicable were excluded for the calculation of the score for the item)

## Supplementary table 4: Quality appraisal scores for each included study (the British Medical Journal Checklist for economic submissions adapted for cost of illness studies; see reference list page 18 to 23 for study ID)

| Reference | ^2^ | ^3^ | ^4^ | ^5^ | ^6^ | ^7^ | ^8^ | ^9^ | ^10^ | ^11^ | ^12^ | ^13^ | ^14^ | ^15^ | ^16^ | ^17^ | ^18^ | ^19^ | ^20^ | ^21^ | ^22^ | ^23^ | ^24^ | ^25^ | ^26^ | ^27^ | ^28^ |
| --- | --- | --- | --- | --- | --- | --- | --- | --- | --- | --- | --- | --- | --- | --- | --- | --- | --- | --- | --- | --- | --- | --- | --- | --- | --- | --- | --- |
| 1. Was a clear definition of the illness given? | 1 | 1 | 1 | 1 | 1 | 0 | 1 | 1 | 1 | 1 | 1 | 1 | 1 | 1 | 1 | p | 1 | 1 | 1 | p | 1 | 1 | 1 | 1 | p | 1 | 1 |
| 2.Were epidemiological sources carefully described? | 1 | 1 | 1 | 1 | 1 | 1 | 1 | 1 | 1 | 1 | 1 | p | 1 | 0 | p | 1 | 1 | 1 | 1 | 1 | 1 | 1 | 1 | 1 | 1 | 1 | 1 |
| 3.Were direct/ indirect costs sufficiently disaggregated? | 0 | 1 | 0 | 1 | 1 | 0 | 0 | n.a | 1 | 0 | 1 | 1 | 1 | 0 | 0 | 0 | 1 | 0 | 0 | 0 | 1 | 1 | 0 | 1 | 1 | 0 | 0 |
| 4.Were activity data sources appropriately described? | 1 | 1 | 1 | 1 | 1 | 1 | 1 | 1 | 1 | p | 1 | 1 | 1 | 0 | 1 | 1 | 1 | 1 | 1 | 1 | 1 | 1 | 1 | 1 | 1 | 1 | 1 |
| 5.Were activity data sources carefully assessed? | 1 | 1 | 1 | 1 | 1 | 0 | 1 | 1 | 1 | p | 1 | 1 | 1 | 1 | 1 | 1 | 1 | 1 | 1 | 1 | 1 | 1 | 1 | 1 | 1 | 1 | 1 |
| 6.Were the sources of all costs values analytically described? | 1 | 1 | 1 | 1 | 1 | 0 | 1 | n.a | 1 | 1 | 1 | n.a | 1 | 1 | 1 | 1 | 1 | 1 | 1 | 1 | 1 | 1 | p | 1 | 1 | 1 | 1 |
| 7. Were unit cost appropriately valued? | 1 | 1 | 1 | 1 | 1 | 1 | 1 | n.a | 0 | 1 | 1 | p | 1 | 1 | 1 | 1 | 1 | 1 | 1 | 1 | 1 | 0 | 1 | 1 | 1 | 1 | 1 |
| 8. Were the methods adopted carefully explained? | p | p | p | 1 | p | 1 | 1 | 1 | 1 | 0 | 1 | 1 | 1 | p | 1 | p | p | 1 | 1 | 1 | 1 | 1 | 1 | 1 | 1 | 1 | 1 |
| 9. Were the major assumptions tested in a sensitivity analysis? | 1 | 1 | 1 | 0 | 0 | 0 | 0 | 1 | 0 | 0 | 0 | 0 | 0 | 0 | 1 | 0 | 1 | 1 | 1 | 0 | 1 | 1 | 1 | 0 | 0 | 0 | 0 |
| 10. Was the presentation of study results consistent with the methodology of the study? | 1 | 1 | 1 | 1 | p | 1 | 1 | 1 | p | 1 | p | 1 | 1 | 0 | 1 | p | p | 1 | 1 | 1 | 1 | 1 | 1 | 1 | 1 | 1 | 1 |
| Total Score by study | **8.5** | **9.5** | **8.5** | **9** | **8** | **5** | **8** | **7** | **7.5** | **6** | **8.5** | **7** | **9** | **4.5** | **8.5** | **6.5** | **9** | **9** | **9** | **7.5** | **10** | **9** | **8.5** | **9** | **8.5** | **8** | **8** |

| Reference | ^29^ | ^30^ | ^31^ | ^32^ | ^33^ | ^34^ | ^35^ | ^36^ | ^37^ | ^38^ | ^39^ | ^40^ | ^41^ | ^42^ | ^43^ | ^44^ | ^45^ | ^46^ | ^47^ | ^48^ | ^49^ | ^50^ | ^51^ | ^52^ | ^53^ |
| --- | --- | --- | --- | --- | --- | --- | --- | --- | --- | --- | --- | --- | --- | --- | --- | --- | --- | --- | --- | --- | --- | --- | --- | --- | --- |
| 1. Was a clear definition of the illness given? | 1 | 1 | 1 | 1 | 1 | p | 1 | p | 1 | 1 | 1 | 1 | 0 | 1 | 1 | 1 | 1 | 1 | 1 | 1 | 1 | 1 | p | p | p |
| 2.Were epidemiological sources carefully described? | 1 | 1 | 1 | 1 | 1 | 1 | 1 | 1 | 1 | 1 | 1 | 1 | 1 | 1 | 1 | 1 | 1 | p | 1 | 1 | 1 | 1 | 1 | 1 | 1 |
| 3.Were direct/ indirect costs sufficiently disaggregated? | 1 | 0 | 0 | 1 | 1 | 1 | 1 | 0 | 0 | 0 | n.a | 0 | 1 | p | 0 | 0 | 1 | 1 | 0 | 1 | 1 | 1 | 1 | 1 | 1 |
| 4.Were activity data sources appropriately described? | 1 | 1 | 1 | 1 | 1 | 1 | 1 | p | 1 | 1 | 1 | 1 | 1 | 1 | 0 | 0 | 1 | 1 | 0 | 1 | 1 | 1 | 1 | 1 | 1 |
| 5.Were activity data sources carefully assessed? | p | 1 | 1 | 1 | 1 | 1 | 1 | p | 1 | 1 | 1 | 1 | p | 1 | 1 | 0 | 1 | 1 | 0 | 1 | 1 | 1 | 1 | 1 | p |
| 6.Were the sources of all costs values analytically described? | 1 | 1 | 1 | 1 | 1 | 1 | 1 | 1 | 1 | 1 | n.a | 1 | p | 1 | 1 | 0 | 1 | 1 | 1 | 1 | 1 | 1 | 1 | 1 | 1 |
| 7. Were unit cost appropriately valued? | 1 | 1 | 1 | 1 | 1 | 0 | 1 | 1 | 0 | p | n.a | 1 | 1 | 1 | 1 | 1 | 1 | 1 | p | 1 | 1 | 1 | 1 | 1 | 1 |
| 8. Were the methods adopted carefully explained? | 1 | p | 1 | p | 1 | 1 | 1 | 1 | 1 | 1 | 1 | 1 | p | 1 | 1 | p | 1 | p | 1 | p | p | p | p | p | 1 |
| 9. Were the major assumptions tested in a sensitivity analysis? | 1 | 1 | 0 | 0 | 0 | 1 | 1 | 1 | 1 | 0 | n.a | 0 | 0 | 0 | 0 | 1 | 1 | 0 | 1 | 1 | 1 | 1 | 1 | 0 | 0 |
| 10. Was the presentation of study results consistent with the methodology of the study? | 1 | 1 | 1 | p | 1 | 1 | 1 | 1 | 1 | 1 | 1 | 1 | p | p | 1 | p | p | 1 | 1 | p | p | p | 1 | 0 | 1 |
| Total Score by study | **9.5** | **8.5** | **8** | **8** | **9** | **8.5** | **10** | **7.5** | **8** | **7.5** | **6** | **8** | **6** | **8** | **7** | **5** | **9.5** | **8** | **6.5** | **9** | **9** | **9** | **9** | **7** | **8** |

| Reference | ^54^ | ^55^ | ^56^ | ^57^ | ^58^ | ^59^ | ^60^ | ^61^ | ^62^ | ^63^ | ^64^ | ^65^ | ^66^ | ^67^ | ^68^ | ^69^ | ^70^ | ^71^ | ^72^ | ^73^ | ^74^ | ^75^ | ^76^ | ^77^ | ^78^ | ^79^ |
| --- | --- | --- | --- | --- | --- | --- | --- | --- | --- | --- | --- | --- | --- | --- | --- | --- | --- | --- | --- | --- | --- | --- | --- | --- | --- | --- |
| 1. Was a clear definition of the illness given? | 1 | 0 | 1 | p | p | p | p | 1 | 0 | p | 1 | 1 | 0 | p | 1 | 1 | 1 | 1 | 1 | p | p | 1 | 1 | 1 | 1 | 1 |
| 2.Were epidemiological sources carefully described? | 1 | 1 | 1 | 1 | n.a | n.a | 1 | 1 | 1 | 1 | 1 | 1 | 1 | 1 | 1 | 1 | 1 | 1 | 1 | 1 | 1 | 1 | 1 | 1 | 1 | 1 |
| 3.Were direct/ indirect costs sufficiently disaggregated? | 1 | 1 | 1 | 1 | 1 | 1 | 1 | 0 | p | 1 | 0 | p | p | 1 | 1 | p | 1 | p | p | 1 | 1 | 1 | 0 | n.a | 0 | 1 |
| 4.Were activity data sources appropriately described? | 1 | p | 1 | 1 | 1 | 1 | 1 | 1 | 0 | 1 | p | 1 | 1 | 1 | 1 | 1 | 1 | 1 | 1 | 1 | 1 | 1 | 0 | 1 | 1 | 1 |
| 5.Were activity data sources carefully assessed? | 1 | p | 1 | 1 | 1 | 1 | 1 | 1 | 0 | 1 | 1 | 1 | 1 | 1 | 1 | 1 | 1 | 1 | 1 | 1 | 0 | 1 | 0 | 1 | 1 | 1 |
| 6.Were the sources of all costs values analytically described? | 1 | 1 | p | 1 | 1 | 1 | 1 | p | p | 1 | 1 | 1 | p | 0 | 1 | 1 | 1 | 1 | 1 | 1 | p | 0 | 0 | n.a | 0 | 1 |
| 7. Were unit cost appropriately valued? | 1 | p | 1 | 1 | 1 | 1 | 1 | 1 | 0 | 0 | 1 | 1 | p | 0 | 1 | 1 | 1 | 1 | 1 | 1 | 0 | 1 | 0 | n.a | 1 | 1 |
| 8. Were the methods adopted carefully explained? | 1 | 0 | 1 | 1 | 1 | 1 | 1 | p | p | 1 | 1 | 1 | 1 | 1 | 1 | 1 | 1 | 1 | 1 | 1 | 1 | 1 | 1 | 1 | 1 | 1 |
| 9. Were the major assumptions tested in a sensitivity analysis? | 0 | 1 | 1 | 1 | 0 | 0 | 1 | 0 | 0 | 0 | 0 | 0 | 0 | 0 | 0 | 0 | 1 | 0 | 0 | 0 | 1 | 0 | 0 | 0 | 1 | 0 |
| 10. Was the presentation of study results consistent with the methodology of the study? | p | 1 | 1 | 1 | 1 | 1 | 1 | 1 | 1 | 1 | 1 | 1 | 1 | 1 | 1 | 1 | p | 1 | 1 | 1 | p | 1 | 1 | 1 | 1 | 1 |
| Total Score by study | **8.5** | **6.5** | **9.5** | **9.5** | **7.5** | **7.5** | **9.5** | **7** | **3.5** | **7.5** | **7.5** | **8.5** | **6.5** | **6.5** | **9** | **8.5** | **9.5** | **8.5** | **8.5** | **8.5** | **6.5** | **8** | **4** | **6** | **8** | **9** |

| Reference | ^80^ | ^81^ | ^82^ | ^83^ | ^84^ | ^85^ | ^86^ | ^87^ | ^88^ | ^89^ | ^90^ | ^91^ | ^92^ | ^93^ | ^94^ | ^95^ | ^96^ | ^97^ | ^98^ | ^99^ | ^100^ | ^101^ | ^102^ | ^103^ | ^104^ |
| --- | --- | --- | --- | --- | --- | --- | --- | --- | --- | --- | --- | --- | --- | --- | --- | --- | --- | --- | --- | --- | --- | --- | --- | --- | --- |
| 1. Was a clear definition of the illness given? | 1 | 1 | 1 | 1 | 1 | 1 | 1 | 1 | 1 | 1 | 1 | 1 | 0 | 1 | 1 | 1 | 1 | 1 | 1 | 1 | p | 1 | 1 | 1 | 0 |
| 2.Were epidemiological sources carefully described? | 1 | 1 | 1 | 1 | 1 | 1 | 1 | 1 | 1 | 1 | 1 | 1 | 1 | 1 | 1 | 1 | 1 | 1 | p | 1 | 1 | p | 1 | 1 | 1 |
| 3.Were direct/ indirect costs sufficiently disaggregated? | 1 | 1 | 1 | 1 | 1 | 1 | 1 | 1 | 1 | 1 | 1 | p | p | 0 | 1 | 1 | 1 | p | 1 | 1 | 1 | 1 | 1 | 1 | 1 |
| 4.Were activity data sources appropriately described? | 1 | 1 | 0 | 1 | p | p | 1 | 1 | 1 | 1 | 1 | p | 1 | p | 1 | p | p | 1 | 1 | 1 | 1 | 1 | 1 | 1 | 1 |
| 5.Were activity data sources carefully assessed? | 1 | 1 | 1 | 1 | 1 | 1 | 1 | 1 | 1 | 1 | 1 | 1 | 1 | 1 | 1 | 1 | 1 | 0 | 1 | 1 | p | 1 | 1 | 1 | 1 |
| 6.Were the sources of all costs values analytically described? | 1 | 1 | 1 | p | 1 | p | 1 | p | 1 | 1 | p | p | 1 | p | p | p | p | p | 1 | p | p | p | 1 | 1 | 1 |
| 7. Were unit cost appropriately valued? | 1 | 1 | 1 | 1 | 1 | 1 | 1 | 1 | 1 | 1 | p | 1 | p | 1 | 1 | p | p | 0 | 1 | 1 | 1 | p | 1 | 1 | 1 |
| 8. Were the methods adopted carefully explained? | 1 | 1 | 1 | 1 | 1 | 1 | 1 | 1 | 1 | 1 | 1 | 1 | 1 | 1 | p | 1 | 1 | 1 | 1 | 1 | 1 | 1 | p | 1 | 1 |
| 9. Were the major assumptions tested in a sensitivity analysis? | 0 | 0 | 0 | 0 | 0 | 0 | 0 | 1 | 0 | 1 | 1 | 0 | 0 | 0 | 0 | 0 | 0 | 0 | 0 | 0 | 1 | 1 | 0 | 0 | 1 |
| 10. Was the presentation of study results consistent with the methodology of the study? | 1 | 1 | 1 | 1 | 1 | 1 | 1 | 1 | 1 | 1 | 1 | 1 | 1 | 1 | 1 | 1 | 1 | p | 1 | 1 | 1 | 1 | 1 | 1 | 1 |
| Total Score by study | **9** | **9** | **8** | **8.5** | **8.5** | **8** | **9** | **9.5** | **9** | **10** | **9** | **7.5** | **7** | **7** | **8** | **7.5** | **7.5** | **5.5** | **8.5** | **8.5** | **8.5** | **8.5** | **8.5** | **9** | **9** |

| Reference | ^105^ | ^106^ | ^107^ | ^108^ | ^109^ | ^110^ | ^111^ | ^112^ | ^113^ | ^114^ | ^115^ | ^116^ | ^117^ | ^118^ | ^119^ | ^120^ | ^121^ | ^122^ | ^123^ | ^124^ |
| --- | --- | --- | --- | --- | --- | --- | --- | --- | --- | --- | --- | --- | --- | --- | --- | --- | --- | --- | --- | --- |
| 1. Was a clear definition of the illness given? | p | 0 | 0 | p | p | 1 | 0 | 1 | 0 | 1 | 1 | 1 | 1 | 1 | 1 | 0 | 1 | 1 | 1 | 1 |
| 2.Were epidemiological sources carefully described? | 1 | 1 | 1 | 1 | 1 | 1 | 1 | 1 | 1 | p | 1 | 1 | 0 | 1 | 1 | 0 | 1 | 1 | 1 | 1 |
| 3.Were direct/ indirect costs sufficiently disaggregated? | 1 | 1 | p | p | p | 0 | 1 | p | p | 1 | 0 | 1 | 1 | n.a | 1 | n.a | 0 | 1 | p | p |
| 4.Were activity data sources appropriately described? | 1 | 0 | 1 | 1 | 0 | p | 1 | 1 | 1 | 1 | 0 | 1 | 1 | 1 | 1 | 1 | 1 | 1 | 1 | 1 |
| 5.Were activity data sources carefully assessed? | 1 | 0 | 1 | 1 | 1 | p | 1 | 1 | 1 | p | 0 | 1 | 1 | 1 | 1 | 0 | 1 | 0 | 0 | 1 |
| 6.Were the sources of all costs values analytically described? | 1 | 1 | 0 | 1 | 0 | 1 | 1 | 1 | p | 1 | 0 | 1 | 1 | 1 | p | p | 1 | 0 | 0 | p |
| 7. Were unit cost appropriately valued? | 1 | 1 | 0 | 1 | 0 | 1 | 1 | 1 | p | 1 | 0 | 1 | 1 | 0 | p | 0 | p | 0 | 0 | 1 |
| 8. Were the methods adopted carefully explained? | 1 | 1 | 1 | 1 | 1 | 1 | 1 | 1 | 1 | 1 | 1 | 1 | 1 | 1 | 1 | 1 | p | p | 1 | 1 |
| 9. Were the major assumptions tested in a sensitivity analysis? | 0 | 1 | 0 | 0 | 0 | 0 | 1 | 1 | 0 | 0 | 0 | 0 | 0 | 1 | 1 | 1 | 0 | 0 | 0 | 0 |
| 10. Was the presentation of study results consistent with the methodology of the study? | 1 | 1 | 1 | 1 | 1 | 1 | 1 | 1 | 1 | 1 | 1 | 1 | 1 | p | p | p | p | p | p | 1 |
| Total Score by study | **8.5** | **7** | **5.5** | **8** | **5** | **7** | **9** | **9.5** | **6.5** | **8** | **4** | **9** | **8** | **7.5** | **8.5** | **4** | **6.5** | **5** | **5** | **8** |

| Reference | ^125^ | ^126^ | ^127^ | ^128^ | ^129^ | ^130^ | ^131^ | ^132^ | ^133^ | ^134^ | ^135^ | ^136^ | ^137^ | ^138^ | ^139^ |
| --- | --- | --- | --- | --- | --- | --- | --- | --- | --- | --- | --- | --- | --- | --- | --- |
| 1. Was a clear definition of the illness given? | 1 | 1 | 0 | 1 | p | 1 | 0 | 1 | 0 | 1 | 0 | 0 | 0 | 0 | 0 |
| 2.Were epidemiological sources carefully described? | 1 | p | 1 | 1 | 1 | 1 | 1 | 1 | 1 | p | n.a | 1 | 1 | p | 1 |
| 3.Were direct/ indirect costs sufficiently disaggregated? | p | 1 | n.a | 1 | 1 | 0 | 1 | p | p | 0 | 1 | 0 | 1 | 1 | 1 |
| 4.Were activity data sources appropriately described? | 1 | p | 1 | 1 | 1 | 1 | p | 1 | 1 | n.a | 1 | 1 | 1 | p | 1 |
| 5.Were activity data sources carefully assessed? | p | 1 | 1 | p | 1 | 0 | 1 | 1 | 1 | 0 | 1 | p | p | 1 | 1 |
| 6.Were the sources of all costs values analytically described? | 1 | p | n.a | 1 | p | 0 | p | p | p | p | 1 | 1 | 1 | 1 | 1 |
| 7. Were unit cost appropriately valued? | p | p | n.a | p | p | 0 | 1 | 0 | 1 | 0 | 1 | 1 | 1 | 1 | 1 |
| 8. Were the methods adopted carefully explained? | p | 1 | 1 | 1 | 1 | 1 | 1 | 0 | p | 1 | 1 | 1 | p | p | 1 |
| 9. Were the major assumptions tested in a sensitivity analysis? | 0 | 0 | 0 | p | 0 | 0 | 1 | 0 | 0 | 0 | 0 | 1 | 0 | 0 | 1 |
| 10. Was the presentation of study results consistent with the methodology of the study? | p | 1 | 1 | p | p | 1 | 1 | p | p | 1 | p | 1 | 1 | p | 1 |
| **Total Score by study** | **6.5** | **7** | **5** | **8** | **7** | **5** | **8** | **5.5** | **6** | **4** | **6.5** | **7.5** | **7** | **6** | **9** |

Each quality criterion was rated as one of: yes (1 point), partial (0.5 points), no (zero points), or not applicable (zero points, plus the item was removed from the denominator).

Total score by study equals to the total number of points allocated as a proportion of the total points applicable for each study.

## Supplementary table 5: Average per person cost estimates for studies reporting costs by severity level. Costs are in 2018 USD purchasing power parity (see reference list page 18 to 23 for study ID)

| **Country** | **Ref** | **Cause** | **Severity** | **Year of cost data** | **Perspective of analysis** | **Unit of Observation** | **in 2018 USD ppp** | | | | |
| --- | --- | --- | --- | --- | --- | --- | --- | --- | --- | --- | --- |
|  |  |  |  |  |  |  | **Direct Costs** | | | **Productivity Losses** | **Informal Care costs** |
|  |  |  |  |  |  |  | **Medical (1)** | **Non- medical (2)** | **Total (1) + (2)** |  |  |
| **High-income North America** | | |  |  |  |  |  |  |  |  |  |
| Canada | ^86^ | Glaucoma | all levels of severity | 2001 | healthcare system | patient | 510.66 | **-** | **-** | **-** | **-** |
|  |  |  | mild |  |  |  | 410.14 | **-** | **-** | **-** | **-** |
|  |  |  | moderate |  |  |  | 514.68 | **-** | **-** | **-** | **-** |
|  |  |  | severe |  |  |  | 612.19 | **-** | **-** | **-** | **-** |
| United States | ^87^ | Glaucoma | all levels of severity | not reported | payer | patient | 2,203.79 | **-** | **-** | **-** | **-** |
|  |  |  | stage 0 |  |  |  | 764.88 | **-** | **-** | **-** | **-** |
|  |  |  | stage 1 |  |  |  | 1,817.05 | **-** | **-** | **-** | **-** |
|  |  |  | stage 2 |  |  |  | 2,166.95 | **-** | **-** | **-** | **-** |
|  |  |  | stage 3 |  |  |  | 2,351.11 | **-** | **-** | **-** | **-** |
|  |  |  | stage 4 |  |  |  | 3,025.14 | **-** | **-** | **-** | **-** |
|  |  |  | stage 5 |  |  |  | 3,082.85 | **-** | **-** | **-** | **-** |
| United States | ^33^ | All causes | moderate VI | 2003 | payer | patient | 461.45 | **-** | **-** | **-** | **-** |
|  |  |  | severe VI |  |  |  | 544.38 | **-** | **-** | **-** | **-** |
|  |  |  | blindness |  |  |  | 317.00 | **-** | **-** | **-** | **-** |
| United States | ^88^ | Glaucoma | glaucoma without visual disability | 2015 | payer | patient | 32,063.34 | **-** | **-** | **-** | **-** |
|  |  |  | glaucoma with visual disability |  |  |  | 32,902.57 | **-** | **-** | **-** | **-** |
| United States | ^117^ | AMD | mild VI | 2009 | societal | patient | 9,598.95 | 8,183.61 | - | - | 3,168.22 |
|  |  |  | moderate VI |  |  |  | 7,380.50 | 25,853.27 | - | - | 1,951.92 |
|  |  |  | severe VI |  |  |  | 11,464.74 | 44,814.90 | - | - | 4,019.06 |
|  |  |  | very severe VI |  |  |  | 13,608.70 | 71,617.94 | - | - | 11,361.15 |
| United States | ^90^ | Glaucoma | controlled IOP; no visual field loss | 2005 | healthcare system | patient | **-** | **-** | 269.42 | **-** | **-** |
|  |  |  | controlled IOP; with visual field loss |  |  |  | **-** | **-** | 807.00 | **-** | **-** |
|  |  |  | uncontrolled IOP; no visual field loss |  |  |  | **-** | **-** | 842.42 | **-** | **-** |
|  |  |  | uncontrolled IOP; with visual field loss |  |  |  | **-** | **-** | 842.42 | **-** | **-** |
| United States | ^84^ | Glaucoma | no vision loss | 2007 | payer | patient | **-** | **-** | 15,736.73 | **-** | **-** |
|  |  |  | moderate |  |  |  | **-** | **-** | 18,307.31 | **-** | **-** |
|  |  |  | severe |  |  |  | **-** | **-** | 22,322.20 | **-** | **-** |
|  |  |  | blindness |  |  |  | **-** | **-** | 2,664.19 | **-** | **-** |
| United States | ^123^ | AMD | visual acuity better than 20/32 | not reported | societal | patient | **-** | **-** | 344.99 | **-** | 276.24 |
|  |  |  | visual acuity 20/32 to better than 20/50 |  |  |  | **-** | **-** | 586.86 | **-** | 1,674.63 |
|  |  |  | visual acuity 20/50 to better than 20/80 |  |  |  | **-** | **-** | 1,451.18 | **-** | 4,450.54 |
|  |  |  | visual acuity 20/80 to better than 20/150 |  |  |  | **-** | **-** | 1,873.53 | **-** | 14,180.35 |
|  |  |  | visual acuity 20/150 to better than 20/250 |  |  |  | **-** | **-** | 1,987.70 | **-** | 20,651.75 |
|  |  |  | visual acuity 20/250 or worse |  |  |  | **-** | **-** | 1,952.10 | **-** | 57,809.18 |
| **Western Europe** | |  |  |  |  |  |  |  |  |  |  |
| Germany | ^83^ | Glaucoma | stage 1 | not reported | hospital | patient | 1,273.58 | **-** | **-** | **-** | **-** |
|  |  |  | stage 2 |  |  |  | 1,542.69 | **-** | **-** | **-** | **-** |
|  |  |  | stage 3 |  |  |  | 1,451.94 | **-** | **-** | **-** | **-** |
|  |  |  | stage 4 |  |  |  | 1,868.12 | **-** | **-** | **-** | **-** |
|  |  |  | stage 5 |  |  |  | 1,489.49 | **-** | **-** | **-** | **-** |
| Germany | ^129^ | Diabetic retinopathy | mild non - proliferative diabetic retinopathy without macular edema (39.5% with impaired vision) | 2002 | payer and societal | patient | **-** | **-** | 336.40 | **-** | **-** |
|  |  |  | moderate non- proliferative diabetic retinopathy without macular edema (63.4% with impaired vision) |  |  |  | **-** | **-** | 420.10 | **-** | **-** |
|  |  |  | severe non - proliferative diabetic retinopathy without macular edema (84.2% with impaired vision) |  |  |  | **-** | **-** | 1,588.66 | **-** | **-** |
|  |  |  | - proliferative diabetic retinopathy without macular edema (87,2% with impaired vision) |  |  |  | **-** | **-** | 1,966.91 | **-** | **-** |
|  |  |  | macular edema and any degree of diabetic retinopathy (100% with impaired vision) |  |  |  | **-** | **-** | 3,364.03 | **-** | **-** |
| Italy | ^81^ | Glaucoma | ocular hypertension | 2003 | healthcare system | patient | 1,025.18 | **-** | **-** | **-** | **-** |
|  |  |  | glaucoma |  |  |  | 1,316.07 | **-** | **-** | **-** | **-** |
|  |  |  | advanced glaucoma |  |  |  | 1,890.68 | **-** | **-** | **-** | **-** |
| Sweden | ^130^ | Diabetic retinopathy | any diabetic retinopathy | 2008 | healthcare system | patient | 84.61 | **-** | **-** | **-** | **-** |
|  |  |  | background retinopathy |  |  |  | 30.55 | **-** | **-** | **-** | **-** |
|  |  |  | proliferative diabetic retinopathy (vision threatening) |  |  |  | 301.99 | **-** | **-** | **-** | **-** |
|  |  |  | maculopathy (vision threatening) |  |  |  | 253.82 | **-** | **-** | **-** | **-** |
|  |  |  | proliferative diabetic retinopathy and maculopathy (vision threatening) |  |  |  | 508.81 | **-** | **-** | **-** | **-** |
| **Latin America and Caribbean** | | |  |  |  |  |  |  |  |  |  |
| Mexico | ^91^ | Glaucoma | mild | not reported | healthcare system | patient | 461.15 | **-** | **-** | **-** | **-** |
|  |  |  | moderate |  |  |  | 951.65 | **-** | **-** | **-** | **-** |
|  |  |  | severe |  |  |  | 579.66 | **-** | **-** | **-** | **-** |
| Brazil | ^92^ | Glaucoma | mild | 2010 | healthcare system | patient | 351.20 | **-** | **-** | **-** | **-** |
|  |  |  | moderate |  |  |  | 415.77 | **-** | **-** | **-** | **-** |
|  |  |  | severe |  |  |  | 448.81 |  |  |  |  |
| **South Asia** | | |  |  |  |  |  |  |  |  |  |
| India | ^93^ | Glaucoma | PAC suspect/PACG- stage 0 | not reported | patient | patient | 23.11 | **-** | **-** | **-** | **-** |
|  |  |  | PAC suspect/PACG- stage 1 |  |  |  | 26.43 | **-** | **-** | **-** | **-** |
|  |  |  | PAC suspect/PACG- stage 2 |  |  |  | 21.97 | **-** | **-** | **-** | **-** |
|  |  |  | PAC suspect/PACG- stage 3 |  |  |  | 31.53 | **-** | **-** | **-** | **-** |
|  |  |  | PAC suspect/PACG- stage 4 |  |  |  | 39.98 | **-** | **-** | **-** | **-** |
| **Southeast Asia, East Asia, and Oceania** | | | |  |  |  |  |  |  |  |  |
| Indonesia | ^133^ | Diabetic retinopathy | clinically significant macular edema | 2017 | healthcare system and societal | patient | **-** | **-** | 1,913.71 | **-** | **-** |
|  |  |  | severe non proliferative diabetic retinopathy and proliferative diabetic retinopathy |  |  |  | **-** | **-** | 695.34 | **-** | **-** |
|  |  |  | advance proliferative diabetic retinopathy |  |  |  | **-** | **-** | 3,336.54 | **-** | **-** |
| Taiwan | ^6^ | All causes | moderate VI | 2010 | governmental | patient | 159.41 | **-** | **-** | **-** | **-** |
|  |  |  | severe VI |  |  |  | 200.50 | **-** | **-** | **-** | **-** |
|  |  |  | blindness |  |  |  | 212.09 | **-** | **-** | **-** | **-** |
| Thailand | ^126^ | AMD | best corrected visual acuity >=20/50 | 2012 | societal | patient | 311.46 | **-** | **-** | **-** | **-** |
|  |  |  | best corrected visual acuity <20/50 -20/160 |  |  |  | 289.20 | **-** | **-** | **-** | **-** |
|  |  |  | best corrected visual acuity <20/160-20/400 |  |  |  | 222.51 | **-** | **-** | **-** | **-** |
|  |  |  | - best corrected visual acuity <20/400 |  |  |  | 438.85 | **-** | **-** | **-** | **-** |

AMD = Age-related macular degeneration; IOP = Intraocular pressure; PAC = primary angle-closure; PACG= primary angle-closure glaucoma

## Supplementary table 6: Average per person direct cost estimates for studies reporting opthalmic and non-opthalmic costs. Costs are in 2018 USD purchasing power parity (see reference list page 18 to 23 for study ID)

| **Country** | **Ref.** | **Cause** | **Year of cost data** | **Perspective of analysis** | **Unit of Observation** | **in 2018 USD ppp** | | | | | | | | |
| --- | --- | --- | --- | --- | --- | --- | --- | --- | --- | --- | --- | --- | --- | --- |
|  |  |  |  |  |  | **Direct Costs -Ophthalmic** | | | **Direct Costs -Non-Ophthalmic** | | | **Direct Costs -Total** | | |
|  |  |  |  |  |  | **Medical (1)** | **Non-**  **medical (2)** | **Total**  **(1) + (2)** | **Medical (1)** | **Non- medical (2)** | **Total**  **(1) + (2)** | **Medical (1)** | **Non-**  **medical (2)** | **Total**  **(1) + (2)** |
| **High-income North America** | |  |  |  |  |  |  |  |  |  |  |  |  |  |
| Canada | ^116^ | AMD | 2005 | societal | patient | 2498.48 | 5984.5 | 8,482.98 | 2,258.49 | - | - | 4,756.97 | 5984.5 | 10,741.47 |
| Canada | ^114^ | AMD | 2005 | societal | patient | - | - | 9,263.48 | 2,450.15 | - | - | - | - | 11,713.63 |
| United States of America | ^124^ | Dry -AMD | 2011 | payer | patient | - | - | 376.41 | - | - | 27,227.95 | - | - | 27,604.36 |
| United States of America | ^124^ | Wet -AMD | 2011 | payer | patient | - | - | 3,121.74 | - | - | 35,542.84 | - | - | 38,664.58 |
| United States of America | ^119^ | AMD | 2008 | payer | patient | 3,826.87 | - | - | 3,199.41 | - | - | 7,026.28 | - | - |
| **Western Europe** | |  |  |  |  |  |  |  |  |  |  |  |  |  |
| Switzerland | ^109^ | AMD | 2014 | payer | patient | 14,723.01 | - | - | 6,321.72 | - | - | 21,044.72 | - | - |
| France | ^114^ | AMD | 2005 | societal | patient | - | - | 9,519.65 | 1,442.96 | - | - | - | - | 10,962.61 |
| Germany | ^114^ | AMD | 2005 | societal | patient | - | - | 17,103.52 | 2,368.32 | - | - | - | - | 19,471.84 |
| Spain | ^114^ | AMD | 2005 | societal | patient | - | - | 8,890.44 | 1,202.36 | - | - | - | - | 10,092.81 |
| Spain | ^110^ | AMD | 2005 | societal | patient | - | - | 8,890.73 | 1,202.45 | - | - | - | - |  |
| United Kingdom | ^114^ | AMD | 2005 | societal | patient | - | - | 7,720.64 | 2,258.49 | - | - | - | - | 9,979.13 |
| United Kingdom | ^57^ | Cataract | 2004 | healthcare system and personal social services | patient | - | - | 3,269.99 | - | - | 2,841.02 | - | - | 6,111.01 |

AMD=Age- related macular degeneration

## Supplementary table 7: Main characteristics of 14 eligible studies identified following a search update on 20 January 2022

| **Country** | **Ref.** | **Cause** | **Study design** | **Perspective of analysis** | **Epidemiological approach** | **Method of resource quantification** | **Type of reported costs** |
| --- | --- | --- | --- | --- | --- | --- | --- |
| **Global** | ^140^ | VI & Blindness | cost of illness | societal | prevalence | top down | productivity losses |
| **High-income North America** | |  |  |  |  |  |  |
| United States | ^141^ | VI & Blindness | cost analysis | health system | prevalence | bottom up | direct costs |
| United States | ^142^ | Cataract | cost analysis | societal | prevalence | bottom up | direct costs |
| United States | ^143^ | Cataract | cost analysis | payer | prevalence | bottom up | direct costs |
| United States | ^144^ | AMD and Diabetic retinopathy | cost of illness | societal | prevalence | top down and bottom up | direct costs + productivity losses + informal care + loss of well being |
| United States | ^145^ | Diabetic retinopathy | cost effectiveness | payer | prevalence | bottom up | direct costs |
| **Western Europe** | |  |  |  |  |  |  |
| Netherlands | ^146^ | Cataract | cost analysis | payer and health care provider | prevalence | bottom up | direct costs |
| Norway | ^147^ | Cataract | cost effectiveness | societal | prevalence | bottom up | direct costs |
| Portugal | ^148^ | Cataract | cost analysis | provider | prevalence | bottom up | direct costs |
| Spain | ^149^ | URE | cost effectiveness | payer and health care provider | incidence | bottom up | direct costs |
| France | ^150^ | Cataract | cost effectiveness | health system | prevalence | bottom up | direct costs |
| **South Asia** | |  |  |  |  |  |  |
| India | ^151^ | Diabetic retinopathy | cost of illness | patient and health care provider | prevalence | bottom up | direct costs |
| India | ^152^ | Uncorrected refractive error | cost-minimisation | provider | prevalence | top down | direct costs |
| **Southeast Asia** | |  |  |  |  |  |  |
| Indonesia | ^153^ | Cataract | cost effectiveness | societal | prevalence | bottom up | direct costs + productivity losses + informal care |

We re-ran the search (outlined on p1 above) on 20 January 2022 to identify eligible papers published since our initial search. The search identified 487 potential studies, we reviewed the full text of 53 and identified the 14 studies summarized above and listed below (reference #140 to 153 below).

## 3.References (#1 is our protocol, #s2 to 139 are our included studies reported in our manuscript and #s140 to 153 are further eligible studies)

**Protocol**

1. Marques AP, Ramke J, Cairns J, et al. Estimating the global cost of vision impairment and its major causes: protocol for a systematic review. *BMJ Open* 2020; **10**(9): e036689.

**Included studies**

2. Chakravarthy U, Biundo E, Saka RO, Fasser C, Bourne R, Little J-A. The Economic Impact of Blindness in Europe. *Ophthalmic epidemiology* 2017; **24**(4): 239-47.

3. Roberts CB, Hiratsuka Y, Yamada M, et al. Economic cost of visual impairment in Japan. *Archives of ophthalmology (Chicago, Ill : 1960)* 2010; **128**(6): 766-71.

4. Eckert KA, Carter MJ, Lansingh VC, et al. A Simple Method for Estimating the Economic Cost of Productivity Loss Due to Blindness and Moderate to Severe Visual Impairment. *Ophthalmic epidemiology* 2015; **22**(5): 349-55.

5. Guan X, Fu M, Lin F, Zhu D, Vuillermin D, Shi L. Burden of visual impairment associated with eye diseases: exploratory survey of 298 Chinese patients. *BMJ open* 2019; **9**(9): e030561.

6. Wang M-T, Ng K, Sheu S-J, Yeh W-S, Lo Y-W, Lee W-J. Analysis of Excess Direct Medical Costs of Vision Impairment in Taiwan. *Value in health regional issues* 2013; **2**(1): 57-63.

7. Awan H, Malik SM, Khan NU. The economic burden of blindness in Pakistan: a socio-economic and policy imperative for poverty reduction strategies. *Indian journal of ophthalmology* 2012; **60**(5): 358-64.

8. Park H-Y, Ryu H, Kang H-Y, Lee H, Kwon J-W. Clinical and Economic Burden of Visual Impairment in an Aging Society of South Korea. *Asia-Pacific journal of public health* 2015; **27**(6): 631-42.

9. Wang X, Lamoureux E, Zheng Y, Ang M, Wong TY, Luo N. Health burden associated with visual impairment in Singapore: the Singapore epidemiology of eye disease study. *Ophthalmology* 2014; **121**(9): 1837-42.

10. Taylor HR, Pezzullo ML, Keeffe JE. The economic impact and cost of visual impairment in Australia. *The British journal of ophthalmology* 2006; **90**(3): 272-5.

11. Taylor HR, Pezzullo ML, Nesbitt SJ, Keeffe JE. Costs of interventions for visual impairment. *American journal of ophthalmology* 2007; **143**(4): 561-5.

12. Chou S-L, Lamoureux E, Keeffe J. Methods for measuring personal costs associated with vision impairment. *Ophthalmic epidemiology* 2006; **13**(6): 355-63.

13. O'Connor PM, Chou S-L, Lamoureux EL, Keeffe JE. Costs of vision impairment in childhood and youth: diary case studies. *Optometry and vision science : official publication of the American Academy of Optometry* 2008; **85**(11): 1106-9.

14. Wong EYH, Chou S-L, Lamoureux EL, Keeffe JE. Personal costs of visual impairment by different eye diseases and severity of visual loss. *Ophthalmic epidemiology* 2008; **15**(5): 339-44.

15. Keeffe JE, Chou S-L, Lamoureux EL. The cost of care for people with impaired vision in Australia. *Archives of ophthalmology (Chicago, Ill : 1960)* 2009; **127**(10): 1377-81.

16. Wright SE, Keeffe JE, Thies LS. Direct costs of blindness in Australia. *Clinical & experimental ophthalmology* 2000; **28**(3): 140-2.

17. Hsueh Y-sA, Brando A, Dunt D, Anjou MD, Boudville A, Taylor H. Cost of close the gap for vision of Indigenous Australians: On estimating the extra resources required. *The Australian journal of rural health* 2013; **21**(6): 329-35.

18. Economics A. Clear focus: The economic impact of of vision loss in Australia in 2009: Melbourne, Australia; 2010.

19. Alva ML, Gray A, Mihaylova B, Leal J, Holman RR. The impact of diabetes-related complications on healthcare costs: new results from the UKPDS (UKPDS 84). *Diabetic medicine : a journal of the British Diabetic Association* 2015; **32**(4): 459-66.

20. Clarke P, Gray A, Legood R, Briggs A, Holman R. The impact of diabetes-related complications on healthcare costs: results from the United Kingdom Prospective Diabetes Study (UKPDS Study No. 65). *Diabetic medicine : a journal of the British Diabetic Association* 2003; **20**(6): 442-50.

21. Lafuma A, Brezin A, Lopatriello S, et al. Evaluation of non-medical costs associated with visual impairment in four European countries: France, Italy, Germany and the UK. *PharmacoEconomics* 2006; **24**(2): 193-205.

22. Chuvarayan Y, Finger RP, Koberlein-Neu J. Economic burden of blindness and visual impairment in Germany from a societal perspective: a cost-of-illness study. *The European journal of health economics : HEPAC : health economics in prevention and care* 2019; (101134867).

23. Pezzullo L, Streatfeild J, Simkiss P, Shickle D. The economic impact of sight loss and blindness in the UK adult population. *BMC health services research* 2018; **18**(1): 63.

24. Meads C, Hyde C. What is the cost of blindness? *The British journal of ophthalmology* 2003; **87**(10): 1201-4.

25. Schakel W, van der Aa HPA, Bode C, Hulshof CTJ, van Rens GHMB, van Nispen RMA. The Economic Burden of Visual Impairment and Comorbid Fatigue: A Cost-of-Illness Study (From a Societal Perspective). *Investigative ophthalmology & visual science* 2018; **59**(5): 1916-23.

26. Lafuma A, Brezin A, Fagnani F, Mimaud V, Mesbah M, Berdeaux G. Nonmedical economic consequences attributable to visual impairment: a nation-wide approach in France. *The European journal of health economics : HEPAC : health economics in prevention and care* 2006; **7**(3): 158-64.

27. Marques AP, Macedo AF, Hernandez-Moreno L, et al. The use of informal care by people with vision impairment. *PloS one* 2018; **13**(6): e0198631.

28. Marques AP, Macedo AF, Lima Ramos P, et al. Productivity Losses and Their Explanatory Factors Amongst People with Impaired Vision. *Ophthalmic epidemiology* 2019; (cg6, 9435674): 1-15.

29. Rein DB, Zhang P, Wirth KE, et al. The economic burden of major adult visual disorders in the United States. *Archives of ophthalmology (Chicago, Ill : 1960)* 2006; **124**(12): 1754-60.

30. Frick KD, Gower EW, Kempen JH, Wolff JL. Economic impact of visual impairment and blindness in the United States. *Archives of ophthalmology (Chicago, Ill : 1960)* 2007; **125**(4): 544-50.

31. Frick KD, Walt JG, Chiang TH, et al. Direct costs of blindness experienced by patients enrolled in managed care. *Ophthalmology* 2008; **115**(1): 11-7.

32. Gordon KD, Cruess AF, Bellan L, Mitchell S, Pezzullo ML. The cost of vision loss in Canada. 1. Methodology. *Can J Ophthalmol* 2011; **46**(4): 310-4.

33. Javitt JC, Zhou Z, Willke RJ. Association between vision loss and higher medical care costs in Medicare beneficiaries costs are greater for those with progressive vision loss. *Ophthalmology* 2007; **114**(2): 238-45.

34. Wittenborn JS, Zhang X, Feagan CW, et al. The economic burden of vision loss and eye disorders among the United States population younger than 40 years. *Ophthalmology* 2013; **120**(9): 1728-35.

35. Gordois A, Cutler H, Pezzullo L, et al. An estimation of the worldwide economic and health burden of visual impairment. *Global public health* 2012; **7**(5): 465-81.

36. Armstrong KL, Jovic M, Vo-Phuoc JL, Thorpe JG, Doolan BL. The global cost of eliminating avoidable blindness. *Indian journal of ophthalmology* 2012; **60**(5): 475-80.

37. Frick KD, Foster A. The magnitude and cost of global blindness: an increasing problem that can be alleviated. *American journal of ophthalmology* 2003; **135**(4): 471-6.

38. Bastawrous A, Suni AV. Thirty Year Projected Magnitude (to 2050) of Near and Distance Vision Impairment and the Economic Impact if Existing Solutions are Implemented Globally. *Ophthalmic Epidemiol* 2019: 1-6.

39. Harrabi H, Aubin M-J, Zunzunegui MV, Haddad S, Freeman EE. Visual difficulty and employment status in the world. *PloS one* 2014; **9**(2): e88306.

40. Zheng Y-F, Pan C-W, Chay J, Wong TY, Finkelstein E, Saw S-M. The economic cost of myopia in adults aged over 40 years in Singapore. *Investigative ophthalmology & visual science* 2013; **54**(12): 7532-7.

41. Lafuma A, Laurendeau C, Lamerain E, Berdeaux G. Economics and attitudes regarding spectacles in daily life: a European perspective. *Ophthalmic epidemiology* 2009; **16**(4): 218-23.

42. Ruiz-Moreno JM, Roura M, en representacion del grupo del estudio M. Cost of myopic patients with and without myopic choroidal neovascularisation. *Archivos de la Sociedad Espanola de Oftalmologia* 2016; **91**(6): 265-72.

43. Vitale S, Cotch MF, Sperduto R, Ellwein L. Costs of refractive correction of distance vision impairment in the United States, 1999-2002. *Ophthalmology* 2006; **113**(12): 2163-70.

44. Mohammadi S-F, Alinia C, Tavakkoli M, Lashay A, Chams H. Refractive surgery: the most cost-saving technique in refractive errors correction. *International journal of ophthalmology* 2018; **11**(6): 1013-9.

45. Angell B, Ali F, Gandhi M, et al. Ready-made and custom-made eyeglasses in India: a cost-effectiveness analysis of a randomised controlled trial. *BMJ open ophthalmology* 2018; **3**(1): e000123.

46. Griffiths UK, Bozzani F, Muleya L, Mumba M. Costs of eye care services: prospective study from a faith-based hospital in Zambia. *Ophthalmic epidemiology* 2015; **22**(1): 43-51.

47. Smith TST, Frick KD, Holden BA, Fricke TR, Naidoo KS. Potential lost productivity resulting from the global burden of uncorrected refractive error. *Bulletin of the World Health Organization* 2009; **87**(6): 431-7.

48. Naidoo KS, Fricke TR, Frick KD, et al. Potential Lost Productivity Resulting from the Global Burden of Myopia: Systematic Review, Meta-analysis, and Modeling. *Ophthalmology* 2019; **126**(3): 338-46.

49. Fricke TR, Holden BA, Wilson DA, et al. Global cost of correcting vision impairment from uncorrected refractive error. *Bulletin of the World Health Organization* 2012; **90**(10): 728-38.

50. Frick KD, Joy SM, Wilson DA, Naidoo KS, Holden BA. The Global Burden of Potential Productivity Loss from Uncorrected Presbyopia. *Ophthalmology* 2015; **122**(8): 1706-10.

51. Eye Care Comparative Effectiveness Research T. Cost-effectiveness of cataract surgery in Japan. *Japanese journal of ophthalmology* 2011; **55**(4): 333-42.

52. Malot J, Combe C, Moss A, Savary P, Hida H, Ligeon-Ligeonnet P. [Cost of cataract surgery in a public hospital]. *Journal francais d'ophtalmologie* 2011; **34**(1): 10-6.

53. Nghiem-Buffet MH, de Pouvourville G, Renard G, Ullern M, Boureau C, Chaine G. [Cost of managing cataracts. Evaluation of traditional hospitalization and ambulatory surgery]. *Presse medicale (Paris, France : 1983)* 2001; **30**(39-40 Pt 1): 1924-6.

54. Fattore G, Torbica A. Cost and reimbursement of cataract surgery in Europe: a cross-country comparison. *Health economics* 2008; **17**(1 Suppl): S71-82.

55. Haynes R, Gale S, Mugford M, Davies P. Cataract surgery in a community hospital outreach clinic: patients' costs and satisfaction. *Social science & medicine (1982)* 2001; **53**(12): 1631-40.

56. Minassian DC, Rosen P, Dart JK, et al. Extracapsular cataract extraction compared with small incision surgery by phacoemulsification: a randomised trial. *The British journal of ophthalmology* 2001; **85**(7): 822-9.

57. Sach TH, Foss AJE, Gregson RM, et al. Falls and health status in elderly women following first eye cataract surgery: an economic evaluation conducted alongside a randomised controlled trial. *The British journal of ophthalmology* 2007; **91**(12): 1675-9.

58. Lundstrom M, Brege KG, Floren I, Roos P, Stenevi U, Thorburn W. Cataract surgery and effectiveness. 1. Variation in costs between different providers of cataract surgery. *Acta ophthalmologica Scandinavica* 2000; **78**(3): 335-9.

59. Stenevi U, Lundstrom M, Thorburn W. The cost of cataract patients awaiting surgery. *Acta ophthalmologica Scandinavica* 2000; **78**(6): 703-5.

60. Brown GC, Brown MM, Menezes A, Busbee BG, Lieske HB, Lieske PA. Cataract surgery cost utility revisited in 2012: a new economic paradigm. *Ophthalmology* 2013; **120**(12): 2367-76.

61. Dave H, Phoenix V, Becker ER, Lambert SR. Simultaneous vs sequential bilateral cataract surgery for infants with congenital cataracts: Visual outcomes, adverse events, and economic costs. *Archives of ophthalmology (Chicago, Ill : 1960)* 2010; **128**(8): 1050-4.

62. Fenter TC, Naslund MJ, Shah MB, Eaddy MT, Black L. The cost of treating the 10 most prevalent diseases in men 50 years of age or older. *The American journal of managed care* 2006; **12**(4 Suppl): S90-8.

63. Kruger SJ, DuBois L, Becker ER, et al. Cost of intraocular lens versus contact lens treatment after unilateral congenital cataract surgery in the infant aphakia treatment study at age 5 years. *Ophthalmology* 2015; **122**(2): 288-92.

64. Schmier JK, Halpern MT, Covert DW, Matthews GP. Evaluation of costs for cystoid macular edema among patients after cataract surgery. *Retina (Philadelphia, Pa)* 2007; **27**(5): 621-8.

65. O'Brien JJ, Gonder J, Botz C, Chow KY, Arshinoff SA. Immediately sequential bilateral cataract surgery versus delayed sequential bilateral cataract surgery: potential hospital cost savings. *Canadian journal of ophthalmology Journal canadien d'ophtalmologie* 2010; **45**(6): 596-601.

66. Arieta CEL, Nascimento MA, Lira RPC, Kara-Jose N. [Waste of medical tests in preoperative evaluation for cataract surgery]. *Cadernos de saude publica* 2004; **20**(1): 303-10.

67. Kara N, Jr., Sirtoli MGGM, Santhiago MR, Parede TRR, Espindola RFd, Carvalho RdS. Phacoemulsification versus extracapsular extraction: governmental costs. *Clinics (Sao Paulo, Brazil)* 2010; **65**(4): 357-61.

68. Saad Filho R, Saad FGL, Freitas LLd. [Cost of phacoemulsification in the national campaign of elective cataract surgery in Itapolis, SP, Brazil]. *Arquivos brasileiros de oftalmologia* 2005; **68**(1): 55-9.

69. Essue BM, Li Q, Hackett ML, et al. A multicenter prospective cohort study of quality of life and economic outcomes after cataract surgery in Vietnam: the VISIONARY study. *Ophthalmology* 2014; **121**(11): 2138-46.

70. Loo C-Y, Kandiah M, Arumugam G, et al. Cost efficiency and cost effectiveness of cataract surgery at the Malaysian Ministry of Health ophthalmic services. *International ophthalmology* 2004; **25**(2): 81-7.

71. Fang J, Wang X, Lin Z, Yan J, Yang Y, Li J. Variation of cataract surgery costs in four different graded providers of China. *BMC public health* 2010; **10**(100968562): 543.

72. Gogate P, Dole K, Ranade S, Deshpande M. Cost of pediatric cataract surgery in Maharashtra, India. *International journal of ophthalmology* 2010; **3**(2): 182-6.

73. Muralikrishnan R, Venkatesh R, Prajna NV, Frick KD. Economic cost of cataract surgery procedures in an established eye care centre in Southern India. *Ophthalmic epidemiology* 2004; **11**(5): 369-80.

74. Singh AJ, Garner P, Floyd K. Cost-effectiveness of public-funded options for cataract surgery in Mysore, India. *Lancet (London, England)* 2000; **355**(9199): 180-4.

75. Ibrahim N, Pozo-Martin F, Gilbert C. Direct non-medical costs double the total direct costs to patients undergoing cataract surgery in Zamfara state, Northern Nigeria: a case series. *BMC health services research* 2015; **15**(101088677): 163.

76. Gradin D, Mundia D. Simultaneous bilateral cataract surgery with IOL implantation in children in Kenya. *Journal of pediatric ophthalmology and strabismus* 2012; **49**(3): 139-44.

77. Polack S, Kuper H, Eusebio C, Mathenge W, Wadud Z, Foster A. The impact of cataract on time-use: results from a population based case-control study in Kenya, the Philippines and Bangladesh. *Ophthalmic epidemiology* 2008; **15**(6): 372-82.

78. Baltussen R, Sylla M, Mariotti SP. Cost-effectiveness analysis of cataract surgery: a global and regional analysis. *Bulletin of the World Health Organization* 2004; **82**(5): 338-45.

79. Economics A. Tunnel Vision:The Economic Impact of Primary Open Angle Glaucoma: Melbourne, Australia; 2011.

80. Hagman J. Comparison of resource utilization in the treatment of open-angle glaucoma between two cities in Finland: is more better? *Acta ophthalmologica* 2013; **91 Thesis 3**(101468102): 1-47.

81. Koleva D, Motterlini N, Schiavone M, Garattini L, Study Group G. Medical costs of glaucoma and ocular hypertension in Italian referral centres: a prospective study. *Ophthalmologica Journal international d'ophtalmologie International journal of ophthalmology Zeitschrift fur Augenheilkunde* 2007; **221**(5): 340-7.

82. Thygesen J, Aagren M, Arnavielle S, et al. Late-stage, primary open-angle glaucoma in Europe: social and health care maintenance costs and quality of life of patients from 4 countries. *Current medical research and opinion* 2008; **24**(6): 1763-70.

83. Traverso CE, Walt JG, Kelly SP, et al. Direct costs of glaucoma and severity of the disease: a multinational long term study of resource utilisation in Europe. *The British journal of ophthalmology* 2005; **89**(10): 1245-9.

84. Bramley T, Peeples P, Walt JG, Juhasz M, Hansen JE. Impact of vision loss on costs and outcomes in medicare beneficiaries with glaucoma. *Archives of ophthalmology (Chicago, Ill : 1960)* 2008; **126**(6): 849-56.

85. Gieser DK, Tracy Williams R, O'Connell W, et al. Costs and utilization of end-stage glaucoma patients receiving visual rehabilitation care: a US multisite retrospective study. *Journal of glaucoma* 2006; **15**(5): 419-25.

86. Iskedjian M, Walker J, Vicente C, et al. Cost of glaucoma in Canada: analyses based on visual field and physician's assessment. *Journal of glaucoma* 2003; **12**(6): 456-62.

87. Lee PP, Walt JG, Doyle JJ, et al. A multicenter, retrospective pilot study of resource use and costs associated with severity of disease in glaucoma. *Archives of ophthalmology (Chicago, Ill : 1960)* 2006; **124**(1): 12-9.

88. Prager AJ, Liebmann JM, Cioffi GA, Blumberg DM. Self-reported Function, Health Resource Use, and Total Health Care Costs Among Medicare Beneficiaries With Glaucoma. *JAMA ophthalmology* 2016; **134**(4): 357-65.

89. Kaplan RI, De Moraes CG, Cioffi GA, Al-Aswad LA, Blumberg DM. Comparative Cost-effectiveness of the Baerveldt Implant, Trabeculectomy With Mitomycin, and Medical Treatment. *JAMA ophthalmology* 2015; **133**(5): 560-7.

90. Rein DB, Wittenborn JS, Lee PP, et al. The cost-effectiveness of routine office-based identification and subsequent medical treatment of primary open-angle glaucoma in the United States. *Ophthalmology* 2009; **116**(5): 823-32.

91. Lazcano-Gomez G, Ramos-Cadena MdLA, Torres-Tamayo M, Hernandez de Oteyza A, Turati-Acosta M, Jimenez-Roman J. Cost of glaucoma treatment in a developing country over a 5-year period. *Medicine* 2016; **95**(47): e5341.

92. Guedes RAP, Guedes VMP, Chaoubah A. Resources use, costs and effectiveness of non-penetrating deep sclerectomy according to glaucoma stage. *Arquivos brasileiros de oftalmologia* 2011; **74**(6): 400-4.

93. Chakravarti T. The Association of Socioeconomic Status with Severity of Glaucoma and the Impacts of Both Factors on the Costs of Glaucoma Medications: A Cross-Sectional Study in West Bengal, India. *Journal of ocular pharmacology and therapeutics : the official journal of the Association for Ocular Pharmacology and Therapeutics* 2018; **34**(6): 442-51.

94. Adio AO, Onua AA. Economic burden of glaucoma in Rivers State, Nigeria. *Clinical ophthalmology (Auckland, NZ)* 2012; **6**(101321512): 2023-31.

95. Studnicka J, Rihova B, Rencova E, et al. Cost and effectiveness of therapy for wet age-related macular degeneration in routine clinical practice. *Ophthalmologica Journal international d'ophtalmologie International journal of ophthalmology Zeitschrift fur Augenheilkunde* 2013; **230**(1): 34-42.

96. Hanemoto T, Hikichi Y, Kikuchi N, Kozawa T. The impact of different anti-vascular endothelial growth factor treatment regimens on reducing burden for caregivers and patients with wet age-related macular degeneration in a single-center real-world Japanese setting. *PloS one* 2017; **12**(12): e0189035.

97. Kume A, Ohshiro T, Sakurada Y, Kikushima W, Yoneyama S, Kashiwagi K. Treatment Patterns and Health Care Costs for Age-Related Macular Degeneration in Japan: An Analysis of National Insurance Claims Data. *Ophthalmology* 2016; **123**(6): 1263-8.

98. Kim S, Park SJ, Byun SJ, Park KH, Suh HS. Incremental economic burden associated with exudative age-related macular degeneration: a population-based study. *BMC health services research* 2019; **19**(1): 828.

99. Spooner KL, Mhlanga CT, Hong TH, Broadhead GK, Chang AA. The burden of neovascular age-related macular degeneration: a patient's perspective. *Clinical ophthalmology (Auckland, NZ)* 2018; **12**(101321512): 2483-91.

100. Saxena N, George PP, Hoon HB, Han LT, Onn YS. Burden of Wet Age-Related Macular Degeneration and Its Economic Implications in Singapore in the Year 2030. *Ophthalmic epidemiology* 2016; **23**(4): 232-7.

101. Athanasakis K, Fragoulakis V, Tsiantou V, Masaoutis P, Maniadakis N, Kyriopoulos J. Cost-effectiveness analysis of ranibizumab versus verteporfin photodynamic therapy, pegaptanib sodium, and best supportive care for the treatment of age-related macular degeneration in Greece. *Clinical therapeutics* 2012; **34**(2): 446-56.

102. Bandello F, Augustin A, Sahel J-A, et al. Association between visual acuity and medical and non-medical costs in patients with wet age-related macular degeneration in France, Germany and Italy. *Drugs & aging* 2008; **25**(3): 255-68.

103. Bonastre J, Le Pen C, Soubrane G, Quentel G. The burden of age-related macular degeneration: results of a cohort study in two French referral centres. *PharmacoEconomics* 2003; **21**(3): 181-90.

104. Dakin HA, Wordsworth S, Rogers CA, et al. Cost-effectiveness of ranibizumab and bevacizumab for age-related macular degeneration: 2-year findings from the IVAN randomised trial. *BMJ open* 2014; **4**(7): e005094.

105. Garattini L, Castelnuovo E, Lanzetta P, et al. Direct medical costs of age-related macular degeneration in Italian hospital ophthalmology departments. A multicenter, prospective 1-year study. *The European journal of health economics : HEPAC : health economics in prevention and care* 2004; **5**(1): 22-7.

106. Grieve R, Guerriero C, Walker J, et al. Verteporfin photodynamic therapy cohort study: report 3: cost effectiveness and lessons for future evaluations. *Ophthalmology* 2009; **116**(12): 2471-2.

107. Reich O, Schmid MK, Rapold R, Bachmann LM, Blozik E. Injections frequency and health care costs in patients treated with aflibercept compared to ranibizumab: new real-life evidence from Switzerland. *BMC ophthalmology* 2017; **17**(1): 234.

108. Schmid MK, Reich O, Blozik E, et al. Outcomes and costs of Ranibizumab and Aflibercept treatment in a health-service research context. *BMC ophthalmology* 2018; **18**(1): 64.

109. Schmid MK, Reich O, Faes L, et al. Comparison of Outcomes and Costs of Ranibizumab and Aflibercept Treatment in Real-Life. *PloS one* 2015; **10**(8): e0135050.

110. Ruiz-Moreno JM, Coco RM, Garcia-Arumi J, Xu X, Zlateva G. Burden of illness of bilateral neovascular age-related macular degeneration in Spain. *Current medical research and opinion* 2008; **24**(7): 2103-11.

111. Ke KM. The direct, indirect and intangible costs of visual impairment caused by neovascular age-related macular degeneration. *The European journal of health economics : HEPAC : health economics in prevention and care* 2010; **11**(6): 525-31.

112. Vottonen P. Anti-vascular endothelial growth factors treatment of wet age-related macular degeneration: from neurophysiology to cost-effectiveness. *Acta ophthalmologica* 2018; **96 Suppl A109**(101468102): 1-46.

113. Weyer-Wendl H, Walter P. Financial burden and quality of life of informal caregivers of patients with wet age-related macular degeneration. *Health economics review* 2016; **6**(1): 37.

114. Cruess AF, Zlateva G, Xu X, et al. Economic burden of bilateral neovascular age-related macular degeneration: multi-country observational study. *PharmacoEconomics* 2008; **26**(1): 57-73.

115. Soubrane G, Cruess A, Lotery A, et al. Burden and health care resource utilization in neovascular age-related macular degeneration: findings of a multicountry study. *Archives of ophthalmology (Chicago, Ill : 1960)* 2007; **125**(9): 1249-54.

116. Cruess A, Zlateva G, Xu X, Rochon S. Burden of illness of neovascular age-related macular degeneration in Canada. *Canadian journal of ophthalmology Journal canadien d'ophtalmologie* 2007; **42**(6): 836-43.

117. Brown MM, Brown GC, Lieske HB, Tran I, Turpcu A, Colman S. Societal costs associated with neovascular Age-related Macular Degeneration in the United States. *Retina (Philadelphia, Pa)* 2016; **36**(2): 285-98.

118. Coleman AL, Yu F. Eye-related medicare costs for patients with age-related macular degeneration from 1995 to 1999. *Ophthalmology* 2008; **115**(1): 18-25.

119. Day S, Acquah K, Lee PP, Mruthyunjaya P, Sloan FA. Medicare costs for neovascular age-related macular degeneration, 1994-2007. *American journal of ophthalmology* 2011; **152**(6): 1014-20.

120. Gower EW, Cassard SD, Bass EB, Schein OD, Bressler NM. A cost-effectiveness analysis of three treatments for age-related macular degeneration. *Retina (Philadelphia, Pa)* 2010; **30**(2): 212-21.

121. Gupta OP, Shienbaum G, Patel AH, Fecarotta C, Kaiser RS, Regillo CD. A treat and extend regimen using ranibizumab for neovascular age-related macular degeneration clinical and economic impact. *Ophthalmology* 2010; **117**(11): 2134-40.

122. Schmier JK, Halpern MT, Covert DW, Delgado J, Sharma S. Impact of visual impairment on service and device use by individuals with age-related macular degeneration (AMD). *Disability and rehabilitation* 2006; **28**(21): 1331-7.

123. Schmier JK, Halpern MT, Covert D, Delgado J, Sharma S. Impact of visual impairment on use of caregiving by individuals with age-related macular degeneration. *Retina (Philadelphia, Pa)* 2006; **26**(9): 1056-62.

124. Schmier JK, Covert DW, Lau EC. Patterns and costs associated with progression of age-related macular degeneration. *American journal of ophthalmology* 2012; **154**(4): 675-81.e1.

125. Yildirim S, Akkin C, Oztas Z, Nalcaci S, Afrashi F, Mentes J. Direct Treatment Costs of Neovascular Age-related Macular Degeneration and Comparison of Gained and/or Preserved Vision with Expenditure. *Turkish journal of ophthalmology* 2018; **48**(1): 27-32.

126. Dilokthornsakul P, Chaiyakunapruk N, Ruamviboonsuk P, et al. Health resource utilization and the economic burden of patients with wet age-related macular degeneration in Thailand. *International journal of ophthalmology* 2014; **7**(1): 145-51.

127. Varano M, Eter N, Winyard S, Wittrup-Jensen KU, Navarro R, Heraghty J. The emotional and physical impact of wet age-related macular degeneration: findings from the wAMD Patient and Caregiver Survey. *Clinical ophthalmology (Auckland, NZ)* 2016; **10**(101321512): 257-67.

128. Economics A. The Economic Impact of Diabetic Eye Disease :a dynamic economic model. Melbourne, Australia: Centre Eye Research Australia, University of Melbourne; 2008.

129. Happich M, Reitberger U, Breitscheidel L, Ulbig M, Watkins J. The economic burden of diabetic retinopathy in Germany in 2002. *Graefe's archive for clinical and experimental ophthalmology = Albrecht von Graefes Archiv fur klinische und experimentelle Ophthalmologie* 2008; **246**(1): 151-9.

130. Heintz E, Wirehn AB, Peebo BB, Rosenqvist U, Levin LA. Prevalence and healthcare costs of diabetic retinopathy: a population-based register study in Sweden. *Diabetologia* 2010; **53**(10): 2147-54.

131. Hutton DW, Stein JD, Glassman AR, Bressler NM, Jampol LM, Sun JK. Five-Year Cost-effectiveness of Intravitreous Ranibizumab Therapy vs Panretinal Photocoagulation for Treating Proliferative Diabetic Retinopathy: A Secondary Analysis of a Randomized Clinical Trial. *JAMA Ophthalmol* 2019: 1-9.

132. Brook RA, Kleinman NL, Patel S, Smeeding JE, Beren IA, Turpcu A. United States comparative costs and absenteeism of diabetic ophthalmic conditions. *Postgraduate medicine* 2015; **127**(5): 455-62.

133. Sasongko MB, Wardhana FS, Febryanto GA, et al. The estimated healthcare cost of diabetic retinopathy in Indonesia and its projection for 2025. *The British journal of ophthalmology* 2019; (azk, 0421041).

134. Prajna VN, Nirmalan PK, Saravanan S, Srinivasan M. Economic analysis of corneal ulcers in South India. *Cornea* 2007; **26**(2): 119-22.

135. Frick KD, Keuffel EL, Bowman RJ. Epidemiological, demographic, and economic analyses: measurement of the value of trichiasis surgery in The Gambia. *Ophthalmic epidemiology* 2001; **8**(2-3): 191-201.

136. Baltussen RMPM, Sylla M, Frick KD, Mariotti SP. Cost-effectiveness of trachoma control in seven world regions. *Ophthalmic epidemiology* 2005; **12**(2): 91-101.

137. Frick KD, Hanson CL, Jacobson GA. Global burden of trachoma and economics of the disease. *The American journal of tropical medicine and hygiene* 2003; **69**(5 Suppl): 1-10.

138. Frick KD, Basilion EV, Hanson CL, Colchero MA. Estimating the burden and economic impact of trachomatous visual loss. *Ophthalmic epidemiology* 2003; **10**(2): 121-32.

139. Redekop WK, Lenk EJ, Luyendijk M, et al. The Socioeconomic Benefit to Individuals of Achieving the 2020 Targets for Five Preventive Chemotherapy Neglected Tropical Diseases. *PLoS neglected tropical diseases* 2017; **11**(1): e0005289.

**Eligible studies 2022**

140. Marques AP, Ramke J, Cairns J, et al. Global economic productivity losses from vision impairment and blindness. *EClinicalMedicine* 2021; **35**.

141. Morse AR, Seiple W, Talwar N, Lee PP, Stein JD. Association of Vision Loss With Hospital Use and Costs Among Older Adults. *JAMA Ophthalmol* 2019; **137**(6): 634-40.

142. Billig JI, Lan WC, Chung KC, Kuo CF, Sears ED. The Increasing Financial Burden of Outpatient Elective Surgery for the Privately Insured. *Ann Surg* 2020; **272**(3): 530-6.

143. Port AD, Nolan JG, Siegel NH, Chen X, Ness SD, Subramanian ML. Combined phaco-vitrectomy provides lower costs and greater area under the curve vision gains than sequential vitrectomy and phacoemulsification. *Graefes Arch Clin Exp Ophthalmol* 2021; **259**(1): 45-52.

144. Moshfeghi AA, Lanitis T, Kropat G, et al. Social Cost of Blindness Due to AMD and Diabetic Retinopathy in the United States in 2020. *Ophthalmic Surg Lasers Imaging Retina* 2020; **51**(4): S6-s14.

145. Holekamp N, Duff SB, Rajput Y, Garmo V. Cost-effectiveness of ranibizumab and aflibercept to treat diabetic macular edema from a US perspective: analysis of 2-year Protocol T data. *J Med Econ* 2020; **23**(3): 287-96.

146. Kruse FM, Groenewoud S, Atsma F, van der Galiën OP, Adang EMM, Jeurissen PPT. Do independent treatment centers offer more value than general hospitals? The case of cataract care. *Health Serv Res* 2019; **54**(6): 1357-65.

147. Hertzberg SNW, Veiby NCBB, Bragadottir R, et al. Cost-effectiveness of the triple procedure – phacovitrectomy with posterior capsulotomy compared to phacovitrectomy and sequential procedures. *Acta Ophthalmologica* 2020; **98**(6): 592-602.

148. Queirós L, Redondo P, França M, et al. Implementing ICHOM standard set for cataract surgery at IPO-Porto (Portugal): clinical outcomes, quality of life and costs. *BMC Ophthalmology* 2021; **21**(1): 119.

149. Balgos M, Piñero DP, Canto-Cerdan M, Alió del Barrio JL, Alió JL. Comparison of the Cost-Effectiveness of SMILE, FS-LASIK, and PRK for Myopia in a Private Eye Center in Spain. *J Refract Surg* 2022; **38**(1): 21-6.

150. Schweitzer C, Brezin A, Cochener B, et al. Femtosecond laser-assisted versus phacoemulsification cataract surgery (FEMCAT): a multicentre participant-masked randomised superiority and cost-effectiveness trial. *Lancet* 2020; **395**(10219): 212-24.

151. Orji A, Rani PK, Narayanan R, Sahoo NK, Das T. The economic burden of diabetic retinopathy care at a tertiary eye care center in South India. *Indian Journal of Ophthalmology* 2021; **69**(3): 666-70.

152. Minakaran N, Morjaria P, Frick KD, Gilbert C. Cost-minimisation Analysis from a Non-inferiority Trial of Ready-Made versus Custom-Made Spectacles for School Children in India. *Ophthalmic Epidemiol* 2021; **28**(5): 383-91.

153. Rochmah TN, Wulandari A, Dahlui M, Ernawaty, Wulandari RD. Cost Effectiveness Analysis Using Disability-Adjusted Life Years for Cataract Surgery. *Int J Environ Res Public Health* 2020; **17**(16).
